# Association between prenatal exposure to alkylphenols and intelligence quotient among preschool children: sex-specific effects

**DOI:** 10.1186/s12940-024-01047-5

**Published:** 2024-02-16

**Authors:** Jinghua Long, Jun Liang, Tao Liu, Huishen Huang, Jiehua Chen, Qian Liao, Lixiang Pang, Kaiqi Yang, Manlin Chen, Qian Chen, Xiaorong Huang, Qihua Zhu, Xiaoyun Zeng, Dongping Huang, Xiaoqiang Qiu

**Affiliations:** 1 Department of Epidemiology and Health Statistics, School of Public Health, Guangxi Medical University, No. 22 Shuangyong Road, Nanning, 530021 Guangxi China; 2 The First Affiliated Hospital of Guangxi Medical University, Nanning, 530021 Guangxi China; 3 Huaihua Center for Disease Control and Prevention, Huaihua, 418000 Hunan China; 4 Department of Sanitary Chemistry, School of Public Health, Guangxi Medical University, No. 22 Shuangyong Road, Nanning, 530021 Guangxi China

**Keywords:** Alkylphenols, Prenatal, Intelligence quotient, Preschool children, Sex-specificity

## Abstract

**Background:**

While prenatal exposure to alkylphenols (APs) has been demonstrated to be associated with neurodevelopmental impairments in animals, the evidence from epidemiological studies remains limited and inconclusive. This study aimed to explore the link between AP exposure during pregnancy and the intelligence quotient (IQ) of preschool children.

**Methods:**

A total of 221 mother-child pairs from the Guangxi Zhuang Birth Cohort were recruited. Nonylphenol (NP), 4-tert-octylphenol (4-T-OP), 4-n-nonylphenol (4-N-NP), and 4-n-octylphenol were measured in maternal serum in early pregnancy. Childhood IQ was evaluated by the Fourth Edition of Wechsler Preschool and Primary Scale of the Intelligence at 3 to 6 years of age. The impact of APs on childhood IQ were evaluated by generalized linear models (GLMs), restricted cubic spline (RCS), and Bayesian kernel machine regression (BKMR).

**Results:**

In GLMs, prenatal exposure to NP and the second tertile of 4-T-OP exhibited an inverse association with full-scale IQ (FSIQ) (*β* = -2.38; 95% CI: -4.59, -0.16) and working memory index (WMI) (*β* = -5.24; 95% CI: -9.58, -0.89), respectively. Prenatal exposure to the third tertile of 4-N-NP showed a positive association with the fluid reasoning index (*β* = 4.95; 95% CI: 1.14, 8.77) in total children, as well as in girls when stratified by sex. A U-shaped relationship between maternal 4-T-OP and WMI was noted in total children and girls by RCS (all *P* nonlinear < 0.05). The combined effect primarily driven by NP, of maternal AP mixtures at concentrations above the 50th percentile exhibited an inverse trend on FSIQ in total children and girls in BKMR.

**Conclusions:**

Prenatal exposure to various APs affects IQ in preschool children, and there may be nonmonotonic and sex-specific effects. Further investigation across the population is required to elucidate the potential neurotoxic effects of APs.

**Supplementary Information:**

The online version contains supplementary material available at 10.1186/s12940-024-01047-5.

## Background

 Alkylphenols (APs) are a significant class of endocrine-disrupting chemicals (EDCs), which are byproducts of the biodegradation of AP polyethoxylates and exhibit estrogenic activity; the two most common types of APs are nonylphenol (NP) and octylphenol (OP) [1]. Due to their widespread and substantial presence in household detergents, plastic products, cosmetics, and personal care items [2], individuals primarily encounter these pollutants through various pathways, including dietary ingestion, skin absorption, and inhalation. Consequently, these pollutants pose a significant threat to health [3].

APs are widely distributed and exhibit environmental stability. They have been detected in biological samples of mothers, including urine, blood, and breast milk [4–7], which leads to exposure in fetuses and infants, with uncertain outcomes. Furthermore, their capacity to traverse the placenta and blood‒brain barrier has generated growing interest in exploring the potential effects of exposure to APs on offspring [6, 8, 9]. In summary, the fetal stage represents a critical developmental period during which exposure to EDCs could potentially have adverse effects on offspring neurodevelopment, leading to long-term consequences [10].

Research has indicated that prenatal exposure to APs may disrupt the thyroid hormone system, trigger an imbalance in oxidative stress, induce inflammation and cellular apoptosis [11, 12], interfere with neuronal DNA replication [13], result in neurotransmitter disorders [14] and impair synaptic plasticity [15]. These effects collectively contribute to potential abnormalities in fetal neurodevelopment. Furthermore, APs are recognized for their estrogenic properties [1], and maternal exposure to APs during pregnancy could potentially disrupt typical neurodevelopment in offspring by exerting neurotoxic effects through anti-androgenic activity [16, 17].

Cognitive function constitutes a crucial element of neurodevelopment. Animal studies have demonstrated a positive association between prenatal exposure to NP and impaired neurodevelopment, as well as learning and memory deficits in offspring [8, 9, 14, 15, 18, 19]. Nevertheless, there is a dearth of epidemiological studies investigating the connection between AP exposure during pregnancy and cognitive outcomes among children. Two prospective birth cohort studies conducted in Taiwan and Spain have investigated the potential connection between prenatal exposure to APs and cognitive function in children [10, 20]. However, neither studies have found statistical significance relationship when adjusted for confounders. Furthermore, no study has explored the potential association between prenatal exposure to 4-n-nonylphenol (4-N-NP) and 4-n-octylphenol (4-N-OP), which are isomers of NP and OP, and the neurocognitive development of children. Thus, the association between prenatal or perinatal exposure to APs and cognitive function remains to be firmly established due to the insufficient evidence available from population studies.

Indeed, in real-world scenarios, humans often encounter simultaneous exposure to multiple APs. Prior investigations have focused on assessing the impacts of individual AP exposure [7, 10, 21]. However, considering the potential synergistic interactions, the combined effects of exposure to chemical mixtures might lead to heightened toxicity [22]. In light of this background, we hypothesize that prenatal exposure to APs is associated with childhood cognitive function. The objective of the present investigation was to investigate the potential links between individual and combined effects of maternal exposure to four APs [NP, 4-N-NP, 4-tert-octylphenol (4-T-OP), and 4-N-OP] during early pregnancy and the IQ of children aged 3 to 6 years, utilizing data from the Guangxi Zhuang Birth Cohort (*GZBC*) in China.

## Methods

### Study design

This research was conducted using a subset of mother-child pairs from the Guangxi Zhuang Birth Cohort (*GZBC*), a prospective population-based study initiated in June 2015, to examine the effects of prenatal environmental exposures on the health outcomes of both mothers and children [23]. Briefly, at baseline, pregnant women who were ≤ 13 gestational weeks, non-assisted reproduction, Zhuang nationality, residents of the study area, and barrier-free communication were included during their initial prenatal care appointment. Maternal blood samples were obtained for laboratory analysis upon completion of a self-administered questionnaire by eligible pregnant women. Children of the eligible pregnant women were followed up from 2016. The present study focused on 221 mother-child pairs with complete maternal serum AP measurement information, singleton pregnancy, without birth defects, children aged 3 to 6 years with IQ scores, and complete covariate information. This research adhered to the principles outlined in the Declaration of Helsinki and received approval from the Ethics and Research Committees of Guangxi Medical University (No.20140305-001). Participants provided written informed consent before being included in the study.

### Serum AP exposure measurements

Fasting venous blood samples of pregnant women were collected and kept at 4 °C. They were then centrifuged at 3500 rpm for 10 min, after which serum samples were extracted and stored at -80 °C until further processing. The procedures for measuring APs have been detailed in previous descriptions [24]. Ultra-performance liquid chromatography-tandem mass spectrometry (UPLCMS, Waters, USA) was used for the quantification analysis of NP, 4-N-NP, 4-T-OP, and 4-N-OP. To prevent contamination from laboratory materials and APs, plastic products were excluded from the sample preparation process. Glass tubes were meticulously cleaned with methanol and ultra-pure water before utilization. Detailed quality control measures were performed as previously described [24]. Table 2 provides the detection rate, limit of detection (LOD), geometric mean, and percentiles (%) of each AP. We assigned sample concentrations below the LOD a value as LOD divided by the square root of 2.

### Childhood IQ assessment

 The Wechsler Intelligence Scales are widely used psychological tests to assess individual’s intellectual abilities [25–27]. It compares an individual’s raw score to the average score of a standardized reference group of the same age and gender during the standardization process to ultimately calculate a standardized IQ score [25–27]. In the present study, childhood IQ was evaluated by the Chinese version of the Wechsler Preschool and Primary Scale of Intelligence, Fourth Edition (WPPSI-IV CN), which possesses well-documented psychometric properties and is widely utilized for evaluating IQ of children aged 2 years 6 months to 6 years [28, 29]. This scale was translated and culturally adapted to conform to Chinese norms, demonstrating an equivalence to the original scales [30]. It comprises five primary subscales that yield standardized scores: the verbal comprehension index (VCI), visual spatial index (VSI), fluid reasoning index (FRI), working memory index (WMI), and processing speed index (PSI). The full-scale intelligence quotient (FSIQ) was calculated based on the five domain subscales [25]. All examiners underwent training supervised by clinical psychologists possessing testing qualifications. The assessments took place in dedicated, private, and tranquil rooms within the project hospitals. The raw data were submitted to a researcher who was blinded to the specifics for entry into the system(King-May, Zhuhai, China) and for calculating the IQ scores of each child. The IQ scores were presented as standardized scores, with an average value of 100 and a standard deviation (SD) normalized to 15 [25].

### Covariates

Sociodemographic covariates were obtained by face-to-face interviews via structured self-administered questionnaire during the first prenatal visit: pre-pregnancy weight (kg) and height (cm), maternal education, alcohol use, passive smoking, folic acid supplementation, parity, household income and diseases history. Detailed reproductive and birth outcome information including gestational hypertension, gestational diabetes mellitus, gestational age, maternal age at delivery, child sex, birth weight and birth length was abstracted from the electronic medical records. Maternal pre-pregnancy body mass index (BMI, kg/m^2^) was computed by dividing weight by the square of height. Confounders that were only associated with outcomes rather than possible consequences of exposure were identified from prior literature [28, 31, 32]. These included maternal age at delivery, maternal pre-pregnancy BMI, maternal education, passive smoking, household income, folic acid supplementation, breastfeeding duration, child sex, and child age at assessment.

### Statistical analysis

Baseline characteristics of the participants were evaluated using independent samples t-tests, Wilcoxon rank sum tests, and chi-square tests. For normally distributed continuous variables, the mean ± SD are provided, while nonnormally distributed variables are expressed as the median and interquartile range (IQR). Categorical variables are displayed as numbers (frequencies). Spearman correlations were used to evaluate the relationships between each pairs of APs, and Pearson correlations were used to test the internal consistency of WPPSI-IV scores. As the AP concentrations in maternal serum were skewed distribution, they were all log10 transformed to treat as continuous variables and used in all models. Covariates, including maternal age at delivery, maternal pre-pregnancy BMI, passive smoking, maternal education, household income, folic acid supplementation, breastfeeding duration, child age, and child sex (except for sex stratification) were adjusted in all models.

Generalized linear models (GLMs) were applied to estimate the relationship between individual maternal AP exposure and childhood IQ, shown as a beta coefficient (β) and its 95% confidence interval (CI). In these models, maternal serum AP concentrations were log10-transformed as continuous variables or tertiled as ordered categorical variables. APs concentration were converted into categorical variables, and an analysis of linear trends for each tertile was conducted by specifying tertiles as integer variables (values 1, 2, and 3 corresponding to the 1st, 2nd, and 3rd tertiles, respectively). Restricted cubic spline (RCS) was used to evaluate the dose‒response connection of each AP and childhood IQ. The RCS model featured three knots positioned at the 10th, 50th, and 90th percentiles of the log10-transformed AP concentrations. The reference point (*β* = 0) was established at the 50th percentile.

We also employed the BKMR model, a nonparametric statistical approach, to estimate the individual effects of each AP, as well as the interactions and combined effects of the AP mixture on childhood IQ [33, 34]. That is, the exposure-response function between each maternal serum APs and childhood IQ was assessed when all the other APs were fixed at their 50th percentiles. Bivariate interactions between each pair of APs were explored when all the other three APs were fixed at their 50th percentile. The cumulative effects of APs on childhood IQ were evaluated by holding all APs at a given quantile compared with their 50th quartile. The combined effects of AP mixtures were performed when four APs were held at their 10th to 90th percentiles, as opposed to being held at their 50th percentile. Posterior inclusion probabilities (PIPs) were calculated to outline the comparative significance of each AP within the mixture concerning its impact on childhood IQ [34]. The BKMR model with 50,000 iterations was implemented by a Markov chain Monte Carlo algorithm.

We conducted two sensitivity analyses. First, as studies have suggested that sex may be a regulatory factor that potentially affects between prenatal environmental chemical exposure and neurodevelopment [28, 31, 32], subgroup analysis with sex stratification was conducted in both single and multi-exposure models to assess whether sex could modify the relationship between maternal AP exposure and childhood IQ. Second, since maternal folic acid supplementation (none, before and during pregnancy) may influence the relationship between prenatal AP exposure and IQ scores among preschool children [35, 36], we ran the GLM again without accounting for maternal folic acid supplementation.

Statistical analyses were performed using R (version 4.1.2). BKMR and RCS models were executed using the bkmr and rms packages in R, respectively. Two-sided *P* < 0.05 was deemed statistically significant.

## Results

### Profile of the enrolled participants

The characteristics of the mother-child pairs are presented in Table 1. The average of maternal age at delivery and pre-pregnancy BMI were 29.31 ± 5.41 years and 20.54 ± 2.97 kg/m^2^, respectively. 39.8% of mothers were passive smokers during pregnancy, and nearly 60.7% of mothers had received a high school education or above. Among the mothers, 48.4% were primiparity and 48.0% were folic acid supplementation during pregnancy. Approximately 77.4% of families reported an annual household income < 150,000 yuan per year. Out of the total participants, 125 (56.6%) were boys and 96 (43.4%) were girls. The average gestational age was 38.64 ± 1.17 weeks, and the mean birth weight was 3123.89 ± 426.13 g. Among the 221 children, 70.1% were breastfed for a minimum of 6 months. The average age of the children at cognitive testing was 4.61 ± 0.64 years. The average of VCI, VSI, WMI, FRI, PSI and FSIQ scores among the children were 85.55 ± 11.35, 90.69 ± 11.62, 90.69 ± 11.62, 100.48 ± 11.24, 99.38 ± 11.31 and 86.90 ± 11.53, respectively.

**Table 1:** Characteristics of the mother-child pairs participants (*n* = 221)

Characteristics	Mean ± SD/number (%)
**Maternal Characteristics**	
Maternal age at delivery (years)	29.31 ± 5.41
Pre-pregnancy BMI (kg/m^2^)	20.54 ± 2.97
Maternal education	
Less than high school	87 (39.4)
high school	43 (19.5)
College graduate or higher	91 (41.2)
Parity	
Primiparity	107 (48.4)
Multipara	114 (51.6)
Folic acid supplement	
None	72 (32.6)
Before pregnancy	43 (19.5)
During pregnancy	106 (48.0)
Passive smoking	
Yes	88 (39.8)
No	133 (60.2)
Household income (yuan/year)	
< 60,000	85 (38.5)
60,000–150,000	86 (38.9)
≥ 150,000	50(22.6)
**Children Characteristics**	
Sex	
Boys	125 (56.6)
Girls	96 (43.4)
Gestational age (weeks)	38.64 ± 1.17
Birth weight (g)	3123.89 ± 426.13
Age (years)	4.61 ± 0.64
Breastfeeding duration (months)	
< 6	66 (29.9)
≥ 6	155 (70.1)
Children’s IQ	
VCI	85.55 ± 11.35
VSI	90.69 ± 11.62
WMI	90.97 ± 13.15
FRI	100.48 ± 11.24
PSI	99.38 ± 11.31
FSIQ	86.90 ± 11.53

*Abbreviations*: *SD* Standard deviation, *BMI* Body mass index, *IQ* Intelligence quotients, *VCI* Verbal comprehension index, *VSI* the visual space index, *FRI* the fluid reasoning index, *WMI* the working memory index, *PSI* Processing speed index, *FSIQ* Full-scale intelligence quotient

### Distributions of maternal serum APs and their associations and the internal consistency of diverse domain scores within the WPPSI-IV assessment

**Fig. 1:**
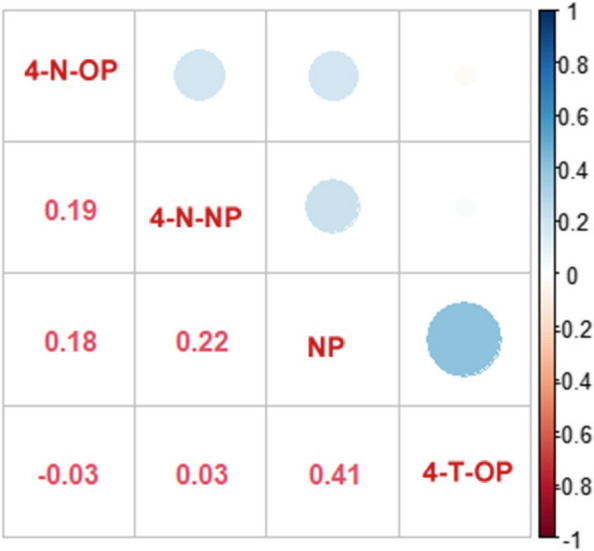
The correlation between the maternal serum APs concentrations among total children. Gradient color from red to blue and circle size represent the correlation coefficient

The distributions of maternal serum APs are presented in Tables S1 and S2. All four APs were detected in maternal serum samples, NP had the highest detection rate (98.2%) while 4-N-OP had the lowest (62.4%) (Table S1). The median concentrations of NP, 4-N-NP, 4-T-OP, and 4-N-OP in 221 maternal serum samples were 95.75 ng/ml, 1.73 ng/ml, 42.92 ng/ml, and 0.84 ng/ml, respectively (Table S2). The AP concentrations were higher among mothers of boys than those in mothers carrying girls, but the difference did not reach statistical significance (*P* > 0.05) (Table S2). The associations between the maternal serum APs are shown in Fig. 1. The Spearman correlation coefficient of each pair of APs was between − 0.03 and 0.41. The internal consistency of various domain scores in the WPPSI-IV is displayed in Table S3. Additionally, scores from different domains displayed significant associations (association coefficients = 0.21 to 0.49, all *P* < 0.01).

### Associations between maternal serum APs and childhood IQ by single-exposure models

Table 2 shows the association between each maternal log10-transformed AP concentration and childhood IQ by GLMs. After adjustment, each unit increase in log10-transformed maternal NP exposure was associated with a 2.38 (95% CI: -4.59,-0.16) decrease in FSIQ. Mothers in the third tertile of log10-transformed 4-N-NP was positively associated with FRI (*β* = 4.95; 95% CI: 1.14, 8.77), while mothers in the second tertile of log10-transformed 4-T-OP was inversely associated with WMI (*β* = -5.24; 95% CI: -9.58,-0.89; *P* for trend = 0.732). Furthermore, Fig. S1 represents the log10-transformed 4-T-OP was nonlinearly related to WMI in total children (*P* nonlinear = 0.017).

**Table 2:** Association between each maternal serum AP concentration (ng/mL; tertile) and child’s intelligence quotient (*n* = 221)

APs	VCI	VSI	WMI	FRI	PSI	FSIQ
	*β* (95%CI)	*β* (95%CI)	*β* (95%CI)	*β* (95%CI)	*β* (95%CI)	*β* (95%CI)
Total						
NP (cont)	-1.78 (-3.95,0.39)	-1.80 (-4.25,0.64)	-2.13 (-4.85,0.59)	0.03 (-3.14,3.21)	-1.08 (-4.40,2.25)	-2.38(-4.59,-0.16)^*^
Tertile 1	Ref	Ref	Ref	Ref	Ref	Ref
Tertile 2	-1.27 (-4.86,2.32)	1.52 (-2.51,5.55)	2.00 (-2.48,6.48)	4.14 (-0.05,8.32)^#^	-0.52 (-5.00,3.96)	0.14 (-3.54,3.82)
Tertile 3	-0.67 (-4.31,2.98)	-0.58 (-4.67,3.51)	-0.36 (-4.91,4.19)	-1.07 (-5.53,3.4)	-1.14 (-5.92,3.64)	-1.12 (-4.85,2.61)
*P* for trend	0.748	0.727	0.817	0.709	0.638	0.533
4-N-NP(cont)	0.91 (-5.85,7.66)	-2.93 (-10.52,4.65)	2.14 (-6.32,10.59)	4.67 (-4.26,13.60)	4.05 (-5.34,13.43)	0.87 (-6.05,7.80)
Tertile 1	Ref	Ref	Ref	Ref	Ref	Ref
Tertile 2	-2.34 (-5.89,1.20)	-1.91 (-5.91,2.09)	-1.93 (-6.39,2.53)	0.03 (-4.24,4.31)	0.83 (-3.74,5.40)	-2.87 (-6.49,0.75)
Tertile 3	0.70 (-2.49,3.89)	-0.29 (-3.89,3.31)	0.12 (-3.89,4.13)	4.95 (1.14,8.77)^*^	2.65 (-1.43,6.73)	1.02 (-2.24,4.27)
*P* for trend	0.708	0.850	0.980	0.014	0.204	0.588
4-T-OP (cont)	-0.12 (-1.59,1.34)	1.10 (-0.54,2.74)	-0.37 (-2.21,1.46)	-0.13 (-1.93,1.67)	-0.26 (-2.15,1.62)	-0.12 (-1.63,1.38)
Tertile 1	Ref	Ref	Ref	Ref	Ref	Ref
Tertile 2	-0.83 (-4.35,2.68)	-2.38 (-6.30,1.53)	-5.24(-9.58,-0.89)^*^	-1.06 (-5.41,3.29)	3.45 (-1.09,7.99)	-1.22 (-4.82,2.38)
Tertile 3	1.23 (-2.35,4.81)	2.04 (-1.94,6.03)	-0.97 (-5.40,3.45)	0.21 (-4.36,4.78)	2.06 (-2.70,6.83)	1.09 (-2.57,4.76)
*P* for trend	0.479	0.285	0.732	0.933	0.388	0.531
4-N-OP(cont)	2.06 (-3.78,7.89)	4.61 (-1.92,11.14)	-1.05 (-8.36,6.26)	4.49 (-2.03,11.00)	1.39 (-5.49,8.26)	3.33 (-2.63,9.30)
Tertile 1	Ref	Ref	Ref	Ref	Ref	Ref
Tertile 2	-0.14 (-3.57,3.28)	0.64 (-3.22,4.50)	-0.81 (-5.11,3.48)	2.44 (-1.86,6.73)	2.61 (-1.90,7.13)	-0.79 (-4.30,2.72)
Tertile 3	0.93 (-2.38,4.23)	0.45 (-3.27,4.17)	-1.32 (-5.46,2.82)	2.76 (-1.34,6.86)	-0.44 (-4.75,3.87)	0.28 (-3.11,3.67)
*P* for trend	0.587	0.805	0.528	0.172	0.911	0.882
Boys						
NP (cont)	-1.64 (-4.84,1.56)	-0.95 (-4.31,2.41)	-1.57 (-5.39,2.26)	-1.40 (-5.65,2.85)	-1.19 (-5.33,2.96)	-1.81 (-5.08,1.47)
Tertile 1	Ref	Ref	Ref	Ref	Ref	Ref
Tertile 2	-0.36 (-5.68,4.97)	2.48 (-3.06,8.03)	1.39 (-4.96,7.73)	5.54 (-0.47,11.54)^#^	0.82 (-5.24,6.89)	0.17 (-5.28,5.62)
Tertile 3	-0.08 (-5.58,5.42)	-0.33 (-6.05,5.40)	-0.62 (-7.18,5.93)	-3.38 (-9.91,3.15)	-3.47 (-10.07,3.13)	-1.20 (-6.82,4.43)
*P* for trend	0.990	0.800	0.789	0.452	0.338	0.641
4-N-NP(cont)	3.41 (-5.77,12.60)	-5.00 (-14.59,4.60)	-1.39 (-12.38,9.59)	2.56 (-11.25,16.38)	11.50(-1.72,24.72)^#^	0.53 (-8.89,9.96)
Tertile 1	Ref	Ref	Ref	Ref	Ref	Ref
Tertile 2	-3.21 (-8.68,2.27)	-4.04 (-9.81,1.73)	-4.41 (-11.00,2.17)	-4.60 (-11.19,1.98)	-1.09 (-7.59,5.41)	-4.59 (-10.16,0.98)
Tertile 3	1.86 (-2.53,6.25)	-1.95 (-6.58,2.68)	-0.65 (-5.93,4.63)	3.32 (-2.01,8.66)	3.95 (-1.32,9.22)	1.67 (-2.80,6.13)
*P* for trend	0.386	0.421	0.831	0.271	0.154	0.440
4-T-OP (cont)	0.49 (-1.63,2.61)	1.53 (-0.67,3.74)	-0.20 (-2.73,2.33)	-0.19 (-2.97,2.58)	-0.65 (-3.36,2.05)	0.29 (-1.88,2.46)
Tertile 1	Ref	Ref	Ref	Ref	Ref	Ref
Tertile 2	-0.16 (-5.25,4.93)	-1.54 (-6.87,3.80)	-5.07 (-11.12,0.98)	-2.37 (-9.04,4.30)	3.35 (-3.08,9.79)	0.22 (-4.95,5.40)
Tertile 3	2.96 (-2.20,8.11)	1.85 (-3.56,7.25)	-1.69 (-7.82,4.44)	-1.05 (-8.24,6.14)	-0.54 (-7.48,6.41)	-0.54 (-5.33,4.24)
*P* for trend	0.249	0.484	0.615	0.783	0.855	0.602
4-N-OP(cont)	2.28 (-6.79,11.36)	2.78 (-6.72,12.29)	-2.36 (-13.2,8.47)	-0.18(-10.58,10.23)	7.64 (-2.35,17.62)	4.38 (-4.89,13.65)
Tertile 1	Ref	Ref	Ref	Ref	Ref	Ref
Tertile 2	1.77 (-3.27,6.81)	1.20 (-4.07,6.48)	-1.00 (-6.99,4.98)	-1.20 (-7.66,5.26)	2.72 (-3.54,8.98)	-0.84 (-6.08,4.40)
Tertile 3	0.42 (-4.25,5.09)	-1.13 (-6.01,3.75)	-3.71 (-9.24,1.83)	0.32 (-5.60,6.25)	-0.55 (-6.29,5.19)	1.35 (-3.95,6.66)
*P* for trend	0.862	0.643	0.185	0.937	0.904	0.821
Girls						
NP (cont)	-1.79 (-4.98,1.41)	-1.75 (-5.63,2.13)	-2.96 (-6.49,0.56)	3.57 (-1.65,8.79)	-1.43 (-7.51,4.65)	-2.62 (-5.79,0.56)
Tertile 1	Ref	Ref	Ref	Ref	Ref	Ref
Tertile 2	-2.36 (-7.62,2.91)	2.40 (-3.99,8.79)	1.86 (-4.00,7.72)	4.97 (-1.28,11.23)	-2.63 (-9.95,4.69)	0.69 (-4.61,5.99)
Tertile 3	-1.39 (-6.76,3.98)	1.19 (-5.33,7.71)	-1.27 (-7.23,4.70)	1.32 (-5.44,8.08)	-0.96 (-8.87,6.96)	-0.86 (-6.26,4.55)
*P* for trend	0.612	0.720	0.664	0.692	0.805	0.749
4-N-NP(cont)	-4.28 (-15.69,7.14)	1.36(-12.53,15.24)	13.48(1.09,25.87)^*^	7.18 (-5.67,20.04)	-0.03 (-14.96,14.91)	1.51 (-9.98,13.00)
Tertile 1	Ref	Ref	Ref	Ref	Ref	Ref
Tertile 2	-1.44 (-6.40,3.52)	-0.99 (-6.99,5.01)	1.57 (-3.92,7.05)	4.57 (-1.38,10.53)	3.06 (-4.05,10.16)	-1.14 (-6.12,3.84)
Tertile 3	-1.16 (-6.20,3.88)	1.74 (-4.36,7.84)	2.59 (-3.04,8.21)	6.66 (0.96,12.36)^*^	1.87 (-4.93,8.68)	-0.17 (-5.23,4.90)
*P* for trend	0.613	0.622	0.350	0.020	0.557	0.905
4-T-OP (cont)	-0.80 (-2.96,1.37)	1.09 (-1.53,3.72)	-0.75 (-3.15,1.66)	0.31 (-2.28,2.89)	0.12 (-2.85,3.10)	-0.6 (-2.77,1.58)
Tertile 1	Ref	Ref	Ref	Ref	Ref	Ref
Tertile 2	-1.41 (-6.49,3.68)	-3.27 (-9.26,2.73)	-4.40 (-10.08,1.28)	-0.08 (-6.25,6.09)	2.67 (-4.40,9.74)	-1.46 (-6.56,3.64)
Tertile 3	-1.33 (-6.81,4.14)	3.71 (-2.73,10.16)	-1.35 (-7.34,4.64)	2.24 (-4.36,8.84)	3.75 (-3.82,11.32)	0.38 (-5.10,5.87)
*P* for trend	0.627	0.267	0.657	0.520	0.311	0.892
4-N-OP(cont)	3.30 (-4.97,11.57)	9.39(-0.46,19.25)^#^	1.08 (-8.13,10.29)	10.30(1.18,19.43)^*^	-3.14 (-14.01,7.73)	4.08 (-4.20,12.37)
Tertile 1	Ref	Ref	Ref	Ref	Ref	Ref
Tertile 2	-2.04 (-7.07,2.98)	0.53 (-5.60,6.66)	-0.97 (-6.61,4.67)	6.78 (0.57,12.98)^*^	2.35 (-5.11,9.81)	-1.68 (-6.74,3.38)
Tertile 3	2.16 (-2.89,7.20)	3.68 (-2.47,9.83)	2.01 (-3.65,7.67)	6.33 (0.17,12.49)^*^	-0.06 (-7.46,7.35)	2.09 (-2.99,7.16)
*P* for trend	0.457	0.248	0.513	0.033	0.956	0.467

*Abbreviations*: *95%CI* 95% Confidence interval, *VCI* Verbal comprehension index, *VSI* the visual space index, *FRI* the fluid reasoning index, *WMI* the working memory index, *PSI* Processing speed index, *FSIQ* full-scale intelligence quotient, *AP* Alkylphenol, *NP* Nonylphenol, *4-N-NP* 4-Nonylphenol, *4-T-OP* 4-tert-octylphenol, *4-N-OP* 4-n-octylphenol

The models were adjusted for maternal age at delivery, maternal pre-pregnancy BMI, passive smoking, maternal education, household income, folic acid supplementation, breastfeeding duration, child age, and child sex

^*^P < 0.05

^#^*P* < 0.10

When stratified by child’s sex, each unit increase in log10-transformed maternal 4-N-NP exposure was associated with a 13.48 (95% CI: 1.09, 25.87) increase in WMI among girls, and mothers in the third tertile of log10-transformed 4-N-NP was positively associated with FRI (*β* = 6.66; 95% CI: 0.96, 12.36) in girls. Moreover, each unit increase in log10-transformed maternal 4-N-OP exposure was associated with a 10.30 (95% CI: 1.18, 19.43) increase in WMI among girls. Among girls, the RCS models also represented there was a dose‒response relationship between log10-transformed 4-N-OP and FRI (*P* overall = 0.048) (Fig. S2), and a U-shaped relationship and between log10-transformed 4-T-OP and WMI (*P* nonlinear = 0.023) (Fig. S2).

The association between maternal AP exposure and childhood IQ remained statistically significant even when not accounting for maternal folic acid supplementation, as well as when stratified by child sex (Table S5).

### Combined effects of the AP mixture by BKMR models

**Fig. 2.**
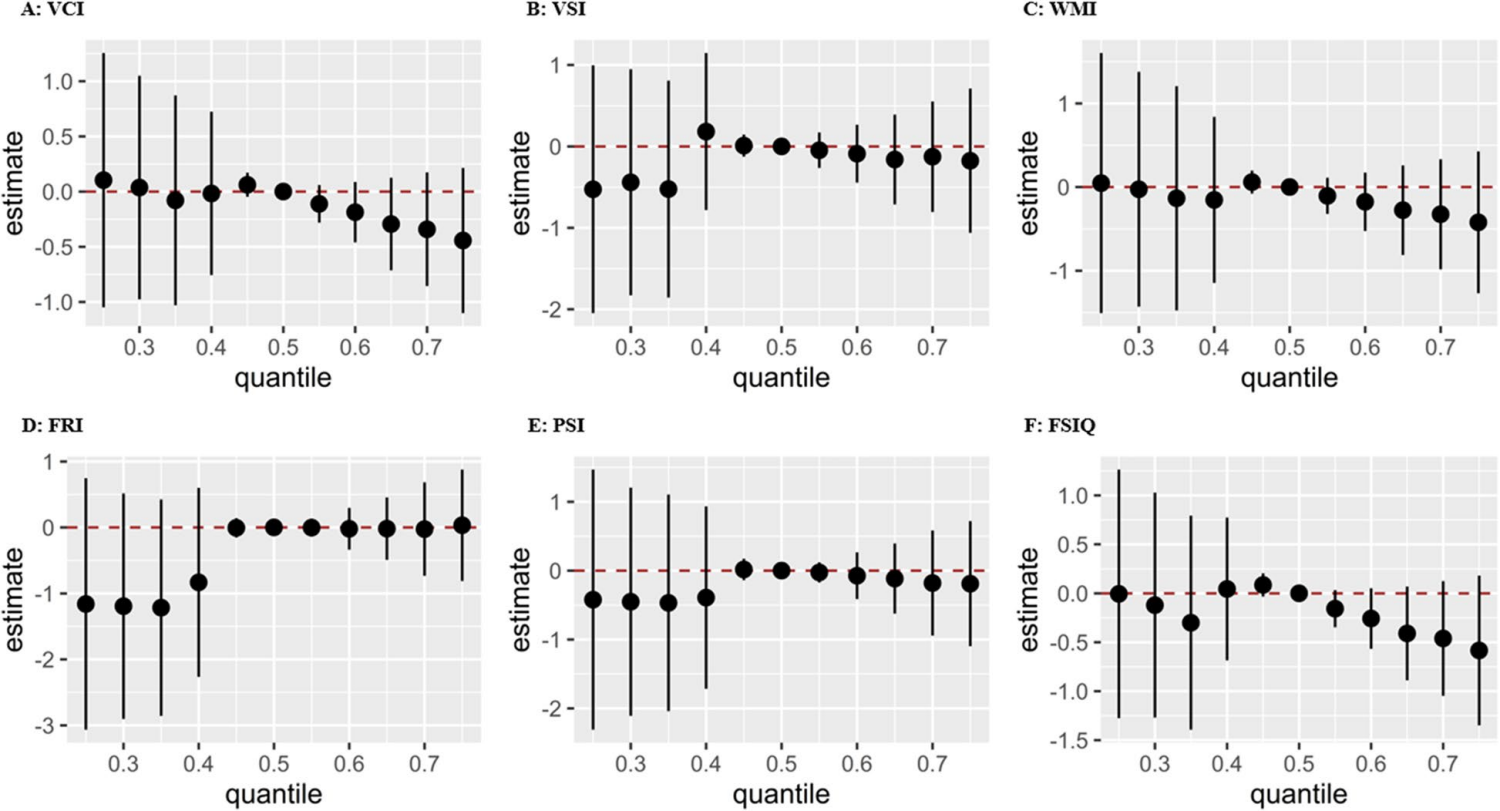
Combined effect of the maternal serum APs on childhood intelligence quotient in total children. APs were log10- transformed. Black circle indicates effect estimates, black vertical lines represent 95% confidence intervals, and red dotted lines represent the null. All models were adjusted for maternal age at delivery, maternal prepregnancy BMI, passive smoking, maternal education, household income, folic acid supplementation, breastfeeding duration, child age, and child sex

**Fig. 3:**
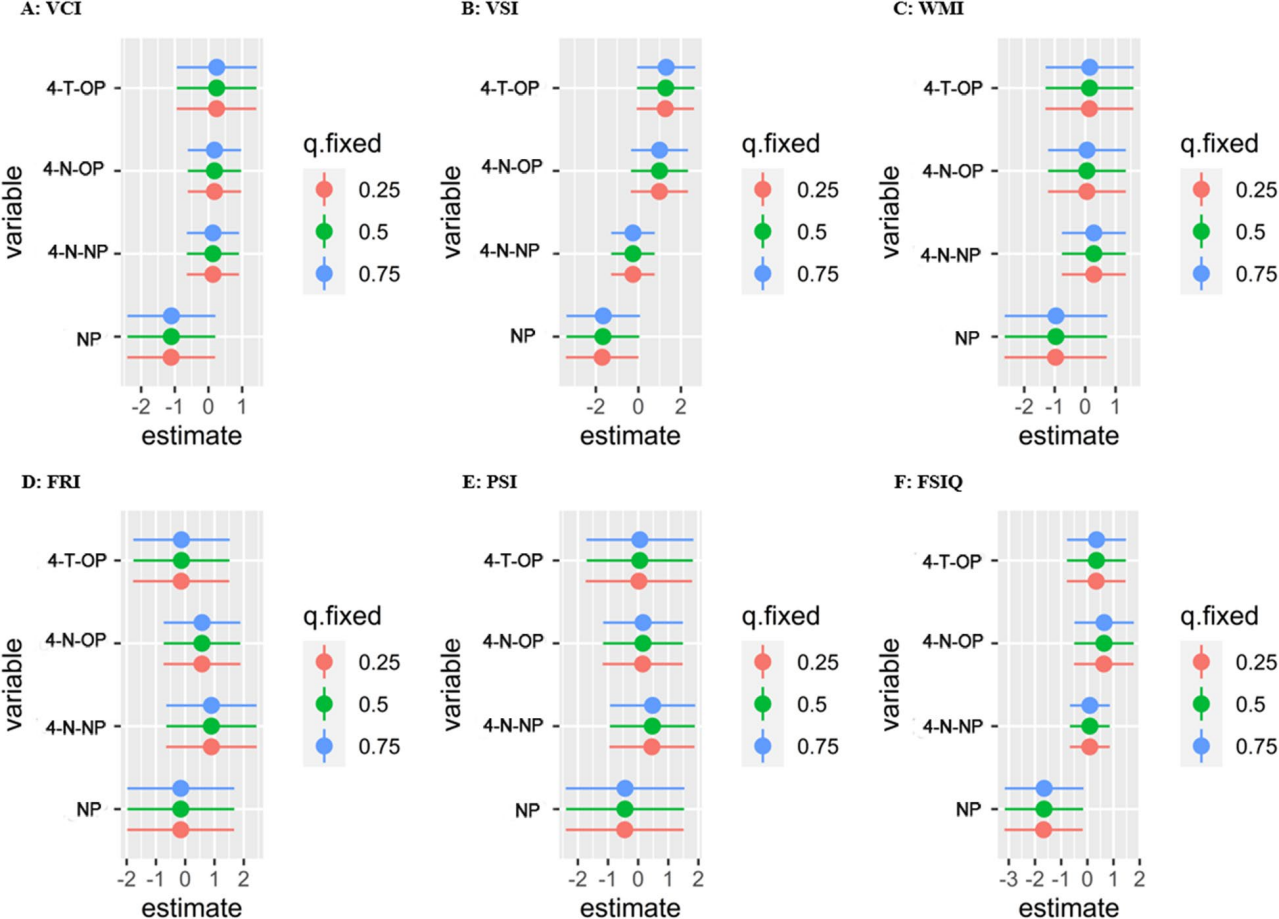
Association (estimates and 95% confidence intervals) of each maternal serum AP with childhood intelligence quotient in total children, when the other APs were fixed at their 25th, 50th, and 75th percentiles. All models were adjusted for maternal age at delivery, maternal prepregnancy BMI, passive smoking, maternal education, household income, folic acid supplementation, breastfeeding duration, child age, and child sex

**Fig. 4:**
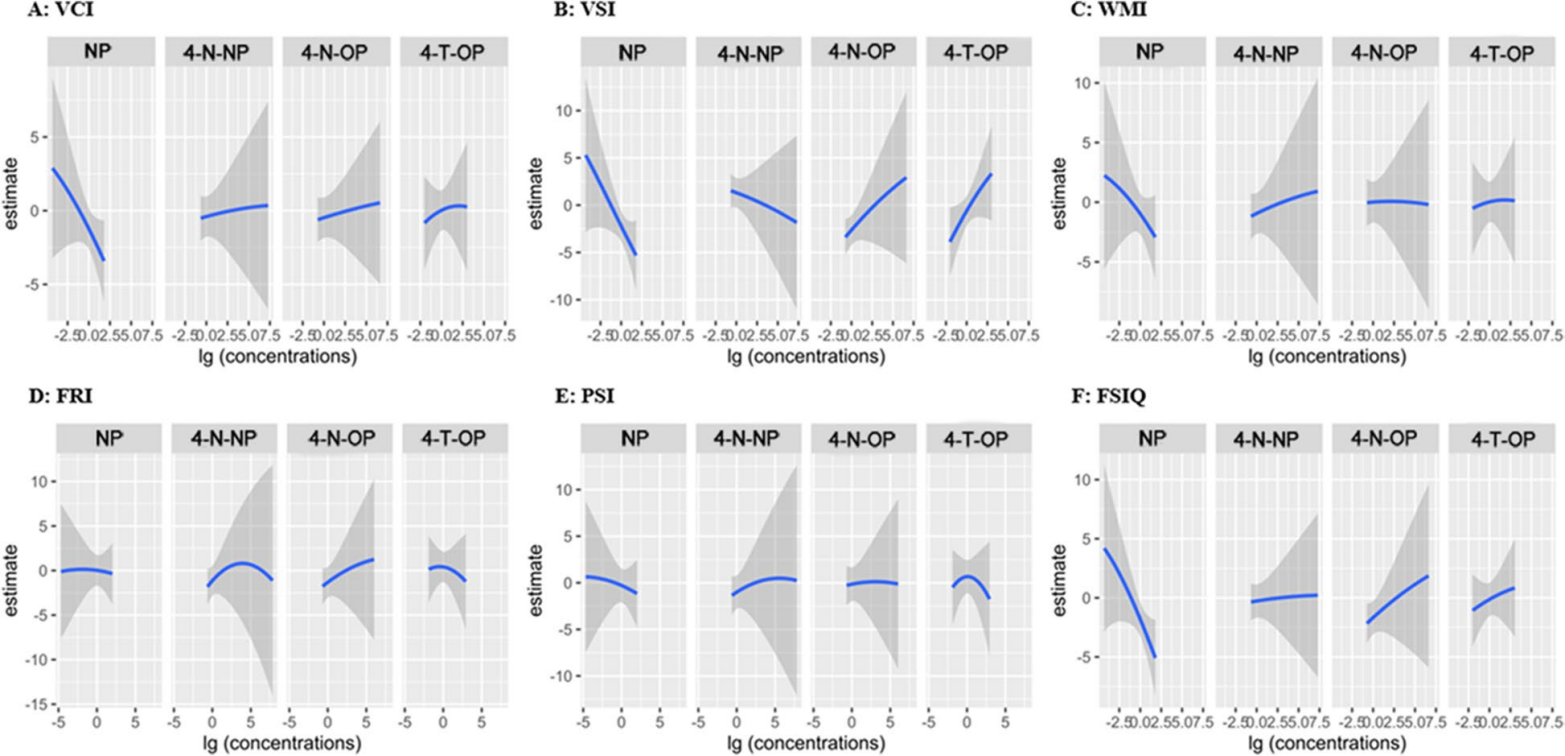
Univariate dose-response function (95% confidence intervals) between the log10-transformed concentrations of per maternal serum APs and childhood intelligence quotient, when fixing the concentrations of other APs at the 50th percentile in total children. Models were adjusted for were adjusted for maternal age at delivery, maternal prepregnancy BMI, passive smoking, maternal education, household income, folic acid supplementation, breastfeeding duration, child age, and child sex

No significant combined effects of the AP mixture on childhood IQ were found (Fig. 2, Figs. S4, S5). Compared with all four APs at their 50th percentile, the combined effect of the AP mixture more than their 50th percentile exhibited a decreasing trend on VCI, VSI, WMI, PSI, and FSIQ in total children (Fig. 2) and girls (Fig. S5). The PIPs rooted in the BKMR model are presented in Table S4. NP had the highest PIPs for VSI, WMI, and FSIQ in total children and in girls. Significant decreases in childhood FSIQ levels were observed when the concentrations of NP increased from the 25th to the 75th percentiles (Fig. 3), however, this change was not significant in either boys or girls (Figs. S6, S7). The univariate dose‒response functions for APs with childhood IQ were generally consistent with the GLM and RCS models (Fig. 4, Figs. S8, S9). For example, when fixing the other APs at their 50th percentile, NP showed a decreasing trend with VCI, VSI, WMI, PSI, and FSIQ in total children (Fig. 4) and girls (Fig. S9). There was no evidence of pairwise interactions between AP exposures and childhood IQ when all the other APs were fixed at their 50th percentile or stratified by sex (Figs. S10-S12).

## Discussion

The present study demonstrates the potential for nonlinear dose‒response relationships and sex-specific associations between prenatal exposure to APs and the IQ of preschool children. Furthermore, prenatal exposure to the AP mixture exceeding the 50th percentile exhibited a decreasing trend in the VCI, VSI, WMI, PSI, and FSIQ scores among total children and among girls. To our knowledge, this study represents one of the initial epidemiological investigations into the impact of maternal serum AP exposure on the IQ of preschool children.

Studies have shown that the detection rate of APs in human, blood samples ranges from 55 to 100% [4–7]. In the current study, the detection rates of NP and 4-T-OP in maternal serum were found to be 98.2% and 91.0%, respectively, which are relatively high. The median concentrations of NP and 4-T-OP in maternal serum were 95.75 ng/mL and 42.93 ng/mL, respectively. Conversely, Huang et al. reported a blood concentration of 14.6 µg/L for NP in pregnant women from Taiwan [6], and Shekhar et al. found blood concentrations of 9.38 µg/L for NP and 5.46 µg/L for OP in pregnant Indian mothers [4]. These levels were significantly below the detection concentrations observed in our study. Although a study from Korea reported elevated blood levels of 89.9 µg/L for NP and 62.2 µg/L for OP [21], their NP levels remained lower than those observed in our study, indicating that pregnant women in the *GZBC* may face elevated potential toxicological risks due to exposure to APs. In addition, elevated maternal NP levels were linked to a higher risk of small for gestational age, shortened birth length, low birth weight, and pubertal development disorders [37–40]. Hence, there is an urgent need for studies investigating the relationship between maternal AP exposure during pregnancy and the growth and development of offspring in Guangxi.

Only two epidemiological studies have investigated the potential link between prenatal exposure to APs and cognitive function in children. Apart from differences in measurement scales, there are also some disparities from our study. The prospective study conducted in Spain revealed that prenatal exposure to APs was linked to weaker performance across various cognitive domains among 5-year-old children. However, these associations did not reach statistical significance [20]. Furthermore, this study constructed an exposure matrix, considering the likelihood of pregnant women encountering various EDCs in their employment during pregnancy, but it did not assess individual-level exposure of the specific APs. In another prospective study carried out in Taiwan, researchers did not observe any potential association between levels of NP and OP in umbilical cord blood and cognitive function among children aged 2 and 7 years. Ultimately, the outcomes of both studies were not impact by the child’s sex, and neither of the studies investigated the combined effects of AP exposure on cognitive development. However, in our study, both single and mixed exposure models were employed to comprehensively investigate the relationship between prenatal AP exposure and the cognitive abilities of preschool children. As a result, we observed some nonmonotonic and sex-specific associations. Given the potential enduring impact of prenatal exposure to EDCs on the developmental trajectory of offspring, our findings propose a potential link between prenatal exposure to APs and adverse cognitive outcomes in preschool children.

Our findings underscore the intricate nature of the connection between prenatal AP exposure and neurodevelopment in preschool children. We observed a negative association between maternal NP and childhood FSIQ scores and a positive association between maternal 4-N-NP and childhood FRI scores. The neuroprotective mechanism of 4-N-NP is not yet clear, but similar to other EDCs [41, 42], APs may act as agonists of peroxisome proliferator-activated receptor (PPAR) and thus influence the PPAR signaling pathway [43, 44], given that receptor agonists of PPAR have been demonstrated to be associated with neuroprotection. Nevertheless, findings have also suggested that APs could potentially induce neurotoxicity through their impact on neuronal DNA replication [13], neuronal differentiation [45], inflammation [11], and injury or apoptosis [13, 46]. These effects could contribute to the impaired neurobehavioral, learning, and memory capabilities observed in rodents [9, 14, 15]. Furthermore, as an isomer of NP, 4-N-NP exhibits distinct exposure sources and levels, which could potentially account for the divergent effects observed in neurocognitive development. However, there is limited research regarding the comparative toxicity between 4-N-NP and NP. To our knowledge, perinatal exposure to 4-N-NP has been associated with behavioral and neurodevelopmental impairments in offspring rats [18]. Indeed, the disparate effects on cognitive function should be interpreted with caution. Nevertheless, given the heterogeneity in associations reported in prior literature, the effects of maternal 4-N-NP exposure on offspring cognitive function should be cautiously interpreted.

Moreover, we noted a nonlinear relationship between maternal 4-T-OP and childhood WMI, and a maternal AP mixture exceeding the 50th percentile exhibited a decreasing trend in VCI, VSI, WMI, PSI, and FSIQ among children. This suggests that the adverse impact of maternal 4-T-OP on childhood WMI intensifies at levels below the median but diminishes at levels above it. Conversely, the detrimental effects of maternal AP mixtures on children’s cognition escalated at concentrations above the median. This phenomenon may be attributed to the disruption of the human endocrine system and gonadal hormones by APs [16, 17, 47, 48], which in turn can disrupt normal neurodevelopmental patterns during early fetal development. Although there are limited studies on the relationship between AP mixtures and child neurodevelopment, the existing research indicates that prenatal exposure to one or multiple EDCs has led to inconsistent effects [31, 32, 49]. This variability could potentially arise from synergistic or antagonistic interactions among different EDCs, resulting in unpredictable neurodevelopmental outcomes. Moreover, genetic variations, timing of exposure, dose‒response relationships, environmental factors, and study designs might further influence the effects of EDCs, contributing to the observed inconsistency in neurodevelopment.

Although the exact biological mechanisms remain uncertain, studies indicate that APs might disrupt essential processes crucial for fetal neurodevelopment. One potential mechanism is that APs may exhibit endocrine-disrupting properties, which could involve interference with the production or homeostasis of the thyroid hormone system [47, 48, 50]. The intrauterine thyroid hormone environment plays a critical role in neurodevelopment, with thyroid hormones being involved in the formation and distribution of somatosensory cortex and hippocampal neurons during early pregnancy [51–53]. Furthermore, maternal hypothyroidism can result in abnormalities in fetal cerebral cortex morphology, neuronal structure, synaptic connections, and axonal myelination [52, 54]. The Generation R Study has also demonstrated a close relationship between maternal thyroid function and hormone levels in early pregnancy and child neurodevelopmental outcomes [55, 56]. In animal experiments, injecting NP into prepubertal goldfish and a combination of NP and OP into lizards destroyed thyroid follicular epithelial cells, and raised thyroid-stimulating hormone levels while reducing triiodothyronine and thyroxine levels [50, 57], indicating that APs, whether alone or combined, can disrupt the hypothalamic-pituitary-thyroid axis, ultimately affecting thyroid hormone levels. Although there is a scarcity of epidemiological studies investigating the link between prenatal AP exposure and thyroid function, animal studies have indicated that NP can traverse the placental barrier and accumulate in the thyroid of offspring [47]. Maternal exposure to NPs during pregnancy and lactation has been linked to adverse effects on the growth and development of pups, including thyroid follicular structure abnormalities [18, 47], as well as learning and memory impairments [15, 18].

Another potential mechanism might be that prenatal exposure to APs may affect child neurodevelopment by oxidative stress and inflammation. Oxidative stress emerges when the body accumulates reactive oxygen species (ROS) beyond the capacity of its antioxidant mechanisms to neutralize them, and finally causes adverse reactions and damage in the organism [58]. Studies have shown the association between prenatal AP exposure and elevated oxidative stress levels during pregnancy [59, 60]. Additionally, increased maternal oxidative stress has been observed to impact the brain development of the offspring [61]. Furthermore, prenatal AP exposure is associated with increased lipid peroxidation during pregnancy [59]. Animal studies have indicated that NP induces the accumulation of ROS in the brain, subsequently leading to lipid peroxidation, which could potentially serve as a catalyst for the apoptotic process [62]. Both lactational and prenatal exposure to NP can activate offspring macrophages and lead to excessive production of proinflammatory cytokines, subsequently, these cytokines travel to the brain through the gut-brain axis, and potentially trigger learning and memory deficits [8, 19]. Other potential mechanisms include neurotransmitter disorder [14], synaptic plasticity impairment [15] and apoptosis [12], all of which can disrupt the normal process of fetal neurodevelopment.

Upon stratifying by child sex, we identified positive associations between prenatal AP exposure and FRI scores in girls, whereas no such associations were observed among boys. These findings imply that sex might modify the connections between prenatal AP exposure and the IQ of preschool children. However, prior studies did not observed any significant associations between prenatal AP exposure and children’s cognitive scores [10, 20]. These differences may arise from variations in children’s age at assessment, neurodevelopmental evaluation scales, or AP exposure levels across different studies. The anti-androgenic properties of APs can result in perturbed androgen levels and abnormal male gonadal development [16, 17]. Furthermore, gonadal hormones not only play a critical role in the sexually dimorphic development of the brain but also hold significant importance in brain remodeling and cognitive function [63]. Animal studies also indicate that greater metabolism of NP in males could potentially be linked to the effects of gender differences in perinatal NP exposure [18, 64]. Hence, further research is warranted to explore the potential influence of gender on the relationship between prenatal AP exposure and child neurodevelopment.

A strength of the study is its prospective examination of prenatal AP exposure and children’s cognitive development. In addition, our study stands out by investigating both 4-N-NP and 4-N-OP, which distinguishes our research from previous studies that focused solely on prenatal exposure to NP and OP in relation to childhood neurodevelopment. Finally, our study is the first to utilize the BKMR model to explore the combined impact of prenatal AP exposure on the IQ of preschool children. Given that chemical exposures often occur as mixtures, it is crucial to investigate their cumulative impacts. Some limitations need to be considered. First, the sample size was relatively limited. Second, some potential confounding factors were not considered in this study, such as maternal occupation, which was included as a confounding factor in other studies [20]. Moreover, we did not assess maternal IQ, although research has suggested that maternal IQ affects offspring’s intelligence level [65]; nevertheless, we did incorporate covariate information concerning maternal education into our analysis. Last, we only measured the AP levels in maternal serum in early pregnancy, which may result in exposure misclassification and uncertainty of the exposure window. Nonetheless, importantly, AP exposure predominantly originates from external environmental sources, including pollutants in food, water, and air. Moreover, the metabolism of APs within the human body varies due to individual differences and the specific types of APs encountered. Hence, maternal serum AP levels during early pregnancy appear to be a suitable biomarker for assessing long-term exposure.

## Conclusions

In conclusion, our findings provide evidence that prenatal exposure to APs during pregnancy might influence the IQ of preschool children, and these effects were modified by the child’s sex. While there might be a nonmonotonic relationship between prenatal exposure to AP mixtures and childhood IQ, our study indeed reveals the neurotoxic effects of certain individual APs, such as NP, upon exposure. Hence, both epidemiological and experimental studies are necessary to further investigate the long-term effects of intrauterine exposure to APs on child neurodevelopment and provide insights into the potential biological mechanisms.

### Supplementary Information

**Additional file 1: Table S1.** Maternal serum APs concentrations (IQR, ng/ml). **Table S2.** Maternal serum AP concentrations (IQR, ng/ml). **Table S3.** Pearson correlation coefficients between scores of five domains among children. **Table S4.** Posterior inclusion probabilities into childhood intelligence quotient of maternal serum APs. **Table S5** Association between each maternal serum APs concentration (ng/mL; tertile) and childhood intelligence quotient in total children (*n*=221) and stratified by child sex (125 boys and 96 girls) when did not adjust for folic acid supplementation in pregnant women. **Fig. S1.** Restricted cubic spline (RCS) models for log10-transformed APs associated with childhood intelligence quotient with knots at the 10th, 50th, and 90th percentiles in total children. **Fig. S2.** Restricted cubic spline (RCS) models for log10-transformed APs associated with childhood intelligence quotient with knots at the 10th, 50th, and 90th percentiles in boys. **Fig. S3.** Restricted cubic spline (RCS) models for log10-transformed APs associated with childhood intelligence quotient with knots at the 10th, 50th, and 90th percentiles in girls. **Fig. S4.** Combined effect of the maternal serum APs on childhood intelligence quotient in boys. **Fig. S5.** Combined effect of the maternal serum APs on childhood intelligence quotient in girls. **Fig. S6.** Association (estimates and 95% confidence intervals) of each maternal serum AP with childhood intelligence quotient in boys, when the other APs were fixed at their 25th, 50th, and 75th percentiles. **Fig. S7.** Association (estimates and 95% confidence intervals) of each maternal serum AP with childhood intelligence quotient in girls, when the other APs were fixed at their 25th, 50th, and 75th percentiles. **Fig. S8.** Univariate dose-response function (95% confidence intervals) between the log10-transformed concentrations of per maternal serum APs and childhood intelligence quotient, when fixing the concentrations of other APs at the 50th percentile in boys. **Fig S9.** Univariate dose-response function (95% confidence intervals) between the log10-transformed concentrations of per maternal serum APs and childhood intelligence quotient, when fixing the concentrations of other APs at the 50th percentile in girls. **Fig. S10.** Bivariate exposure-response function between each pair of maternal serum APs and childhood intelligence quotient, when fixing the other APs at their 25th, 50th, and 75th percentiles in total children. **Fig. S11.** Bivariate exposure-response function between each pair of maternal serum APs and childhood intelligence quotient, when fixing the other APs at their 25th, 50th, and 75th percentiles in boys. **Fig. S12.** Bivariate exposure-response function between each pair of maternal serum APs and childhood intelligence quotient, when fixing the other APs at their 25th, 50th, and 75th percentiles in girls.

## Data Availability

The datasets used and analyzed during the current study are available from the corresponding author on reasonable request.

## Abbreviations

APs: Alkylphenols
BMI: Body mass index
BKMR: Bayesian kernel machine regression
CI: Confidence interval
EDCs: Endocrine-disrupting chemicals
4-N-NP: 4-N-nonylphenol
4-N-OP: 4-N-octylphenol
4-T-OP: 4-Tert-octylphenol
FRI: Fluid reasoning index
GLMs: Generalized linear models
GZBC: Guangxi Zhuang Birth Cohort
IQ: Intelligence quotient
IQR: Interquartile range
LOD: Limit of detection
NP: Nonylphenol
OP: Octylphenol
PPAR: Peroxisome proliferator-activated
PIPs: Posterior inclusion probabilities
PSI: Processing speed index
RCS: Restricted cubic spline
ROS: Reactive oxygen species
SD: Standard deviation
UPLCMS: Ultra-performance liquid chromatography-tandem mass spectrometry
VCI: Verbal comprehension index
VSI: Visual spatial index
WMI: Working memory index
WPPSI: Wechsler Preschool and Primary Scale of Intelligence

## References

[1] Asimakopoulos AG, Thomaidis NS, Koupparis MA (2012). Recent trends in biomonitoring of bisphenol A, 4-t-octylphenol, and 4-nonylphenol. Toxicol Lett.

[2] Raecker T, Thiele B, Boehme RM, Guenther K (2011). Endocrine disrupting nonyl- and octylphenol in infant food in Germany: considerable daily intake of nonylphenol for babies. Chemosphere.

[3] Hsieh CY, Yang L, Kuo WC, Zen YP (2013). Efficiencies of freshwater and estuarine constructed wetlands for phenolic endocrine disruptor removal in Taiwan. Sci Total Environ.

[4] Shekhar S, Sood S, Showkat S, Lite C, Chandrasekhar A, Vairamani M, Barathi S, Santosh W (2017). Detection of phenolic endocrine disrupting chemicals (EDCs) from maternal blood plasma and amniotic fluid in Indian population. Gen Comp Endocrinol.

[5] Azzouz A, Rascón AJ, Ballesteros E (2016). Simultaneous determination of parabens, alkylphenols, phenylphenols, bisphenol A and triclosan in human urine, blood and breast milk by continuous solid-phase extraction and gas chromatography-mass spectrometry. J Pharm Biomed Anal.

[6] Huang YF, Wang PW, Huang LW, Yang W, Yu CJ, Yang SH, Chiu HH, Chen ML (2014). Nonylphenol in pregnant women and their matching fetuses: placental transfer and potential risks of infants. Environ Res.

[7] Shen Y, Dong YM, Lu Q, Xu J, Wu YT, Yun SS, Ren ML (2016). Phenolic environmental estrogens in urine and blood plasma from women with uterine leiomyoma: epidemiological survey. J Obstet Gynaecol Res.

[8] Gu W, Wang Y, Qiu Z, Dong J, Wang Y, Chen J (2018). Maternal exposure to nonylphenol during pregnancy and lactation induces microglial cell activation and pro-inflammatory cytokine production in offspring hippocampus. Sci Total Environ.

[9] Kazemi S, Khalili-Fomeshi M, Akbari A, Kani SNM, Ahmadian SR, Ghasemi-Kasman M (2018). The correlation between nonylphenol concentration in brain regions and resulting behavioral impairments. Brain Res Bull.

[10] Lin CC, Chien CJ, Tsai MS, Hsieh CJ, Hsieh WS, Chen PC (2017). Prenatal phenolic compounds exposure and neurobehavioral development at 2 and 7years of age. Sci Total Environ.

[11] Zhang YQ, Mao Z, Zheng YL, Han BP, Chen LT, Li J, Li F (2008). Elevation of inducible nitric oxide synthase and cyclooxygenase-2 expression in the mouse brain after chronic nonylphenol exposure. Int J Mol Sci.

[12] Li S, Jiang Z, Chai W, Xu Y, Wang Y (2019). Autophagy activation alleviates nonylphenol-induced apoptosis in cultured cortical neurons. Neurochem Int.

[13] Kudo C, Wada K, Masuda T, Yonemura T, Shibuya A, Fujimoto Y, Nakajima A, Niwa H, Kamisaki Y (2004). Nonylphenol induces the death of neural stem cells due to activation of the caspase cascade and regulation of the cell cycle. J Neurochem.

[14] Jie Y, Pan W, Wenxia Y, Feng G, Liting H, Wenmei L, Jie X (2017). The effects of gestational and lactational exposure to Nonylphenol on c-jun, and c-fos expression and learning and memory in hippocampus of male F(1) rat. Iran J Basic Med Sci.

[15] Li M, You M, Li S, Qiu Z, Wang Y (2019). Effects of maternal exposure to nonylphenol on learning and memory in offspring involve inhibition of BDNF-PI3K/Akt signaling. Brain Res Bull.

[16] Xu LC, Sun H, Chen JF, Bian Q, Qian J, Song L, Wang XR (2005). Evaluation of androgen receptor transcriptional activities of bisphenol A, octylphenol and nonylphenol in vitro. Toxicology.

[17] Lee HJ, Chattopadhyay S, Gong EY, Ahn RS, Lee K (2003). Antiandrogenic effects of bisphenol A and nonylphenol on the function of androgen receptor. Toxicol Sci.

[18] Couderc M, Gandar A, Kamari A, Allain Y, Zalouk-Vergnoux A, Herrenknecht C, Le Bizec B, Mouneyrac C, Poirier L (2014). Neurodevelopmental and behavioral effects of nonylphenol exposure during gestational and breastfeeding period on F1 rats. Neurotoxicology.

[19] Che X, Fang Y, You M, Xu Y, Wang Y (2020). Exposure to nonylphenol in early life increases pro-inflammatory cytokines in the prefrontal cortex: involvement of gut-brain communication. Chemico-Biol Interact.

[20] Ish J, Symanski E, Gimeno Ruiz de Porras D, Casas M, Delclos GL, Guxens M, Ibarluzea JM, Iñiguez C, Lertxundi A, Rebagliato M (2022). Maternal occupational exposure to chemicals and child cognitive function. Pediatr Res.

[21] Jung H, Hong Y, Lee D, Pang K, Kim Y (2013). The association between some endocrine disruptors in human plasma and the occurrence of congenital hypothyroidism. Environ Toxicol Pharmacol.

[22] Carpenter DO, Arcaro K, Spink DC (2002). Understanding the human health effects of chemical mixtures. Environ Health Perspect.

[23] Liang J, Liu S, Liu T, Yang C, Wu Y, Jennifer Tan HJ, Wei B, Ma X, Feng B, Jiang Q (2020). Association of prenatal exposure to bisphenols and birth size in Zhuang ethnic newborns. Chemosphere.

[24] Jiang Q, Liu R, Liu T, Liang J, Wu Y, Feng B, Liu S, Li H, Pan D, Qiu X (2022). Relationship between exposure of alkylphenols in serum of pregnant women during early pregnancy and adverse birth outcomes. Environ Sci Pollut Res Int.

[25] Watkins MW, Beaujean AA (2014). Bifactor structure of the Wechsler preschool and primary scale of intelligence–fourth edition. School Psychol Quarterly.

[26] Watkins MW, Smith LG (2013). Long-term stability of the Wechsler intelligence scale for children–fourth edition. Psychol Assess.

[27] Denney DA, Ringe WK, Lacritz LH (2015). Dyadic short forms of the wechsler adult intelligence scale-IV. Arch Clin Neuropsychol.

[28] Zhu YD, Wu XY, Yan SQ, Huang K, Tong J, Gao H, Xie Y, Tao SM, Ding P, Zhu P (2020). Domain- and trimester-specific effect of prenatal phthalate exposure on preschooler cognitive development in the Ma’anshan Birth Cohort (MABC) study. Environ Int.

[29] Wang H, Luo F, Zhang Y, Yang X, Zhang S, Zhang J, Tian Y, Zheng L (2023). Prenatal exposure to perfluoroalkyl substances and child intelligence quotient: evidence from the Shanghai birth cohort. Environ Int.

[30] Li YQZJ. Wechsler Preschool and Primary Scale of Intelligence (Chinese), fourth ed. Series King-May: Zhuhai, Guangdong; 2014.

[31] van den Dries MA, Guxens M, Spaan S, Ferguson KK, Philips E, Santos S, Jaddoe VWV, Ghassabian A, Trasande L, Tiemeier H (2020). Phthalate and Bisphenol exposure during pregnancy and offspring nonverbal IQ. Environ Health Perspect.

[32] Spratlen MJ, Perera FP, Lederman SA, Rauh VA, Robinson M, Kannan K, Trasande L, Herbstman J (2020). The association between prenatal exposure to perfluoroalkyl substances and childhood neurodevelopment. Environ Pollution (Barking Essex: 1987).

[33] Bobb JF, Valeri L, Claus Henn B, Christiani DC, Wright RO, Mazumdar M, Godleski JJ, Coull BA (2015). Bayesian kernel machine regression for estimating the health effects of multi-pollutant mixtures. Biostatistics (Oxford England).

[34] Bobb JF, Claus Henn B, Valeri L, Coull BA (2018). Statistical software for analyzing the health effects of multiple concurrent exposures via bayesian kernel machine regression. Environ Health.

[35] McNulty H, Rollins M, Cassidy T, Caffrey A, Marshall B, Dornan J, McLaughlin M, McNulty BA, Ward M, Strain JJ (2019). Effect of continued folic acid supplementation beyond the first trimester of pregnancy on cognitive performance in the child: a follow-up study from a randomized controlled trial (FASSTT offspring trial). BMC Med.

[36] Caffrey A, McNulty H, Rollins M, Prasad G, Gaur P, Talcott JB, Witton C, Cassidy T, Marshall B, Dornan J (2021). Effects of maternal folic acid supplementation during the second and third trimesters of pregnancy on neurocognitive development in the child: an 11-year follow-up from a randomised controlled trial. BMC Med.

[37] Huang YF, Pan WC, Tsai YA, Chang CH, Chen PJ, Shao YS, Tsai MS, Hou JW, Lu CA, Chen ML (2017). Concurrent exposures to nonylphenol, bisphenol A, phthalates, and organophosphate pesticides on birth outcomes: a cohort study in Taipei, Taiwan. Sci Total Environ.

[38] Tsai MS, Chang CH, Tsai YA, Liao KW, Mao IF, Wang TH, Hwang SM, Chang YJ, Chen ML (2013). Neonatal outcomes of intrauterine nonylphenol exposure–a longitudinal cohort study in Taiwan. Sci Total Environ.

[39] Chang CH, Chen ML, Liao KW, Tsai YA, Mao IF, Wang TH, Hwang SM, Chang YJ, Tsai MS (2013). The association between maternal nonylphenol exposure and parity on neonatal birth weight: a cohort study in Taiwan. Chemosphere.

[40] Chen ML, Lee HY, Chuang HY, Guo BR, Mao IF (2009). Association between nonylphenol exposure and development of secondary sexual characteristics. Chemosphere.

[41] Bility MT, Thompson JT, McKee RH, David RM, Butala JH, Vanden Heuvel JP, Peters JM (2004). Activation of mouse and human peroxisome proliferator-activated receptors (PPARs) by phthalate monoesters. Toxicol Sci.

[42] Gao P, Wang L, Yang N, Wen J, Zhao M, Su G, Zhang J, Weng D (2020). Peroxisome proliferator-activated receptor gamma (PPARγ) activation and metabolism disturbance induced by bisphenol A and its replacement analog bisphenol S using in vitro macrophages and in vivo mouse models. Environ Int.

[43] Kourouma A, Keita H, Duan P, Quan C, Bilivogui KK, Qi S, Christiane NA, Osamuyimen A, Yang K (2015). Effects of 4-nonylphenol on oxidant/antioxidant balance system inducing hepatic steatosis in male rat. Toxicol Rep.

[44] Pereira-Fernandes A, Demaegdt H, Vandermeiren K, Hectors TL, Jorens PG, Blust R, Vanparys C (2013). Evaluation of a screening system for obesogenic compounds: screening of endocrine disrupting compounds and evaluation of the PPAR dependency of the effect. PLoS One.

[45] Nishimura Y, Nagao T, Fukushima N (2014). Long-term pre-exposure of pheochromocytoma PC12 cells to endocrine-disrupting chemicals influences neuronal differentiation. Neurosci Lett.

[46] Pretorius E, Bornman MS, Marx J, Smit E, van der Merwe CF (2006). Ultrastructural effects of low dosage endocrine disrupter chemicals on neural cells of the chicken embryo model. Horm Metabol Res.

[47] Wang L, Xu J, Zeng F, Fu X, Xu W, Yu J (2019). Influence of nonylphenol exposure on basic growth, development, and thyroid tissue structure in F1 male rats. Peer J.

[48] Wang L, Guo M, Feng G, Wang P, Xu J, Yu J (2021). Effects of chronic exposure to nonylphenol at environmental concentration on thyroid function and thyroid hyperplasia disease in male rats. Toxicology.

[49] Qian X, Li J, Xu S, Wan Y, Li Y, Jiang Y, Zhao H, Zhou Y, Liao J, Liu H (2019). Prenatal exposure to phthalates and neurocognitive development in children at two years of age. Environ Int.

[50] He Y, Yang J, Huang S, Liu R, Liu H, Zheng D, Huang Q, Yang Y, Liu C (2019). Protective effect of mulberry crude extract against nonylphenol-induced thyroid disruption by inhibiting the activity of deiodinase in rats. Gen Comp Endocrinol.

[51] de Escobar GM, Obregón MJ, del Rey FE (2004). Maternal thyroid hormones early in pregnancy and fetal brain development. Best Pract Res Clin Endocrinol Metab.

[52] Lavado-Autric R, Ausó E, García-Velasco JV, Arufe Mdel C, Escobar del Rey F, Berbel P (2003). Morreale De Escobar G: early maternal hypothyroxinemia alters histogenesis and cerebral cortex cytoarchitecture of the progeny. J Clin Investig.

[53] Ausó E, Lavado-Autric R, Cuevas E, Del Rey FE, Morreale De Escobar G, Berbel P (2004). A moderate and transient deficiency of maternal thyroid function at the beginning of fetal neocorticogenesis alters neuronal migration. Endocrinology.

[54] Pathak A, Sinha RA, Mohan V, Mitra K, Godbole MM (2011). Maternal thyroid hormone before the onset of fetal thyroid function regulates reelin and downstream signaling cascade affecting neocortical neuronal migration. Cerebral Cortex (New York, NY: 1991).

[55] Jansen TA, Korevaar TIM, Mulder TA, White T, Muetzel RL, Peeters RP, Tiemeier H (2019). Maternal thyroid function during pregnancy and child brain morphology: a time window-specific analysis of a prospective cohort. Lancet Diabetes Endocrinol.

[56] Modesto T, Tiemeier H, Peeters RP, Jaddoe VW, Hofman A, Verhulst FC, Ghassabian A (2015). Maternal mild thyroid hormone insufficiency in early pregnancy and Attention-Deficit/Hyperactivity disorder symptoms in children. JAMA Pediatr.

[57] Sciarrillo R, Di Lorenzo M, Valiante S, Rosati L, De Falco M (2021). OctylPhenol (OP) alone and in combination with NonylPhenol (NP) alters the structure and the function of thyroid gland of the Lizard Podarcis Siculus. Arch Environ Contam Toxicol.

[58] Pizzino G, Irrera N, Cucinotta M, Pallio G, Mannino F, Arcoraci V, Squadrito F, Altavilla D, Bitto A (2017). Oxidative stress: Harms and benefits for Human Health. Oxidative Med Cell Longev.

[59] Wang PW, Chen ML, Huang LW, Yang W, Wu KY, Huang YF (2015). Nonylphenol exposure is associated with oxidative and nitrative stress in pregnant women. Free Radic Res.

[60] Huang YF, Wang PW, Huang LW, Lai CH, Yang W, Wu KY, Lu CA, Chen HC, Chen ML (2017). Prenatal nonylphenol and bisphenol A exposures and inflammation are determinants of Oxidative/Nitrative stress: a Taiwanese cohort study. Environ Sci Technol.

[61] Akhtar F, Rouse CA, Catano G, Montalvo M, Ullevig SL, Asmis R, Kharbanda K, Maffi SK (2017). Acute maternal oxidant exposure causes susceptibility of the fetal brain to inflammation and oxidative stress. J Neuroinflamm.

[62] Mao Z, Zheng YL, Zhang YQ (2010). Behavioral impairment and oxidative damage induced by chronic application of nonylphenol. Int J Mol Sci.

[63] Cohen-Bendahan CC, van de Beek C, Berenbaum SA (2005). Prenatal sex hormone effects on child and adult sex-typed behavior: methods and findings. Neurosci Biobehav Rev.

[64] Green T, Swain C, Van Miller JP, Joiner RL (2003). Absorption, bioavailability, and metabolism of para-nonylphenol in the rat. Regul Toxicol Pharmacol.

[65] Andersson HW, Sommerfelt K, Sonnander K, Ahlsten G (1996). Maternal child-rearing attitudes, IQ, and socioeconomic status as related to cognitive abilities of five-year-old children. Psychol Rep.

