# Supplementary material for: Association between prenatal exposure to alkylphenols and intelligence quotient among preschool children: sex-specific effects
Source: Environ Health. 2024 Feb 16;23:21. doi: 10.1186/s12940-024-01047-5 (PMC10870542; doi:10.1186/s12940-024-01047-5)
Supplement: Supplementary file 1 — Additional file 1: Table S1. Maternal serum APs concentrations (IQR, ng/ml). Table S2. Maternal serum AP concentrations (IQR, ng/ml). Table S3. Pearson correlation coefficients between scores of five domains among children. Table S4. Posterior inclusion probabilities into childhood intelligence quotient of maternal serum APs. Table S5 Association between each maternal serum APs concentration (ng/mL; tertile) and childhood intelligence quotient in total children (n=221) and stratified by child sex (125 boys and 96 girls) when did not adjust for folic acid supplementation in pregnant women. Fig. S1. Restricted cubic spline (RCS) models for log10-transformed APs associated with childhood intelligence quotient with knots at the 10th, 50th, and 90th percentiles in total children. Fig. S2. Restricted cubic spline (RCS) models for log10-transformed APs associated with childhood intelligence quotient with knots at the 10th, 50th, and 90th percentiles in boys. Fig. S3. Restricted cubic spline (RCS) models for log10-transformed APs associated with childhood intelligence quotient with knots at the 10th, 50th, and 90th percentiles in girls. Fig. S4. Combined effect of the maternal serum APs on childhood intelligence quotient in boys. Fig. S5. Combined effect of the maternal serum APs on childhood intelligence quotient in girls. Fig. S6. Association (estimates and 95% confidence intervals) of each maternal serum AP with childhood intelligence quotient in boys, when the other APs were fixed at their 25th, 50th, and 75th percentiles. Fig. S7. Association (estimates and 95% confidence intervals) of each maternal serum AP with childhood intelligence quotient in girls, when the other APs were fixed at their 25th, 50th, and 75th percentiles. Fig. S8. Univariate dose-response function (95% confidence intervals) between the log10-transformed concentrations of per maternal serum APs and childhood intelligence quotient, when fixing the concentrations of other APs at the 50th percentile in [file 12940_2024_1047_MOESM1_ESM.docx]

**Supplemental Information**

**Association between prenatal exposure to alkylphenols and intelligence quotient among preschool children: Sex-specific effects**

**Table of Contents**

**Table S1** Maternal serum APs concentrations (IQR, ng/ml).

**Table S2** Maternal serum AP concentrations (IQR, ng/ml).

**Table S3** Pearson correlation coefficients between scores of five domains among children.

**Table S4** Posterior inclusion probabilities into childhood intelligence quotient of maternal serum APs.

**Table S5** Association between each maternal serum APs concentration (ng/mL; tertile) and childhood intelligence quotient in total children (n=221) and stratified by child sex (125 boys and 96 girls) when did not adjust for folic acid supplementation in pregnant women.

**Fig. S1** Restricted cubic spline (RCS) models for log10-transformed APs associated with childhood intelligence quotient with knots at the 10th, 50th, and 90th percentiles in total children.

**Fig. S2** Restricted cubic spline (RCS) models for log10-transformed APs associated with childhood intelligence quotient with knots at the 10th, 50th, and 90th percentiles in boys.

**Fig. S3** Restricted cubic spline (RCS) models for log10-transformed APs associated with childhood intelligence quotient with knots at the 10th, 50th, and 90th percentiles in girls.

**Fig. S4** Combined effect of the maternal serum APs on childhood intelligence quotient in boys.

**Fig. S5** Combined effect of the maternal serum APs on childhood intelligence quotient in girls.

**Fig. S6** Association (estimates and 95% confidence intervals) of each maternal serum AP with childhood intelligence quotient in boys, when the other APs were fixed at their 25th, 50th, and 75th percentiles. .

**Fig. S7** Association (estimates and 95% confidence intervals) of each maternal serum AP with childhood intelligence quotient in girls, when the other APs were fixed at their 25th, 50th, and 75th percentiles. .

**Fig. S8** Univariate dose-response function (95% confidence intervals) between the log10-transformed concentrations of per maternal serum APs and childhood intelligence quotient, when fixing the concentrations of other APs at the 50th percentile in boys.

**Fig S9** Univariate dose-response function (95% confidence intervals) between the log10-transformed concentrations of per maternal serum APs and childhood intelligence quotient, when fixing the concentrations of other APs at the 50th percentile in girls.

**Fig. S10** Bivariate exposure-response function between each pair of maternal serum APs and childhood intelligence quotient, when fixing the other APs at their 25th, 50th, and 75th percentiles in total children.

**Fig. S11** Bivariate exposure-response function between each pair of maternal serum APs and childhood intelligence quotient, when fixing the other APs at their 25th, 50th, and 75th percentiles in boys .

**Fig. S12** Bivariate exposure-response function between each pair of maternal serum APs and childhood intelligence quotient, when fixing the other APs at their 25th, 50th, and 75th percentiles in girls.

**Table S1** Maternal serum AP concentrations (ng/mL)

| APs | Detection rate (%) | LOD | Geometric mean | Percentiles | | | | |
| --- | --- | --- | --- | --- | --- | --- | --- | --- |
|  |  |  |  | P5 | P25 | P50 | P75 | P95 |
| NP | 98.2 | 0.135 | 79.353 | 7.155 | 45.371 | 95.749 | 204.747 | 525.376 |
| 4-N-NP | 76.5 | 1.718 | 1.666 | 1.215 | 1.215 | 1.733 | 1.781 | 3.461 |
| 4-T-OP | 91.0 | 0.439 | 30.806 | 0.310 | 12.334 | 42.925 | 90.183 | 1002.104 |
| 4-N-OP | 62.4 | 0.787 | 0.834 | 0.556 | 0.556 | 0.843 | 0.952 | 1.665 |

Abbreviations: LOD, limit of detection; APs, Alkylphenols; NP, Nonylphenol; 4-N-NP, 4-nonylphenol; 4-T-OP, 4-tert-octylphenol; 4-N-OP, 4-n-octylphenol.

**Table S2** Maternal serum AP concentrations (IQR, ng/ml)

| APs | Total (n =221) | Mother of boys (n = 125) | Mother of girls (n = 96) | *P* value |
| --- | --- | --- | --- | --- |
| NP | 95.75 (45.72,204.73) | 100.19 (44.35,209.05) | 88.27 (45.98,202.20) | 0.622 |
| 4-N-NP | 1.73 (1.32,1.78) | 1.73 (1.22,1.79) | 1.73 (1.22,1.78) | 0.610 |
| 4-T-OP | 42.92 (12.38,89.03) | 43.44 (8.22,101.31) | 40.66 (16.37,82.40) | 0.751 |
| 4-N-OP | 0.84 (0.63,0.95) | 0.85 (0.56,0.95) | 0.83 (0.56,0.95) | 0.725 |

Abbreviations: APs, Alkylphenols; NP, Nonylphenol; 4-N-NP, 4-Nonylphenol; 4-T-OP, 4-tert-Octylphenol; 4-N-OP, 4-n-Octylphenol.

**Table S3** Pearson correlation coefficients between scores of five domains among children

|  | VCI^1^ | VSI^1^ | WMI^1^ | FRI^2^ | PSI^2^ |
| --- | --- | --- | --- | --- | --- |
| VCI | 1.00 | 0.49*** | 0.46*** | 0.32*** | 0.39*** |
| VSI |  | 1.00 | 0.46*** | 0.37*** | 0.23** |
| WMI |  |  | 1.00 | 0.29*** | 0.21** |
| FRI |  |  |  | 1.00 | 0.25** |
| PSI |  |  |  |  | 1.00 |

1: n=221; 2: n=170.

Abbreviations: VCI, verbal comprehension index; VSI, the visual space index; FRI, the fluid reasoning index; WMI, the working memory index; PSI, processing speed index.

**: *P* <0.01; ***: *P* <0.001

**Table S4** Posterior inclusion probabilities into childhood intelligence quotient of maternal serum APs.

| Variables | VCI | VSI | WMI | FRI | PSI | FSIQ |
| --- | --- | --- | --- | --- | --- | --- |
| Total |  |  |  |  |  |  |
| NP | **0.035** | **0.127** | **0.096** | 0.031 | 0.026 | **0.154** |
| 4-N-NP | 0.008 | 0.013 | 0.008 | 0.081 | 0.041 | 0.010 |
| 4-N-OP | 0.005 | 0.018 | 0.014 | 0.020 | 0.024 | 0.014 |
| 4-T-OP | 0.033 | 0.038 | 0.030 | **0.092** | **0.172** | 0.020 |
| Boys |  |  |  |  |  |  |
| NP | 0.047 | 0.032 | **0.042** | 0.033 | 0.162 | 0.033 |
| 4-N-NP | 0.042 | 0.049 | 0.039 | 0.038 | 0.209 | 0.032 |
| 4-N-OP | 0.019 | 0.024 | 0.038 | 0.028 | 0.222 | 0.020 |
| 4-T-OP | **0.056** | **0.084** | **0.042** | **0.115** | **0.253** | **0.044** |
| Girls |  |  |  |  |  |  |
| NP | 0.018 | **0.106** | **0.030** | 0.225 | 0.062 | **0.052** |
| 4-N-NP | 0.009 | 0.028 | 0.021 | 0.247 | 0.078 | 0.010 |
| 4-N-OP | 0.013 | 0.085 | 0.020 | **0.281** | 0.078 | 0.018 |
| 4-T-OP | **0.123** | 0.049 | 0.026 | 0.226 | **0.245** | 0.030 |

Abbreviations: VCI, verbal comprehension index; VSI, the visual space index; FRI, the fluid reasoning index; WMI, the working memory index; PSI, processing speed index; FSIQ, full-scale intelligence quotient; APs, Alkylphenols; NP, Nonylphenol; 4-N-NP, 4-nonylphenol; 4-T-OP, 4-tert-octylphenol; 4-N-OP, 4-n-octylphenol.

The models were adjusted for maternal age at delivery, maternal pre-pregnancy BMI, passive smoking, maternal education, household income, breastfeeding duration, child age, and child sex (except for sex stratification). All APs were log10-transformed as continuous variables.

**Table S5** Association between each maternal serum APs concentration (ng/mL; tertile) and childhood intelligence quotient in total children (n=221) and stratified by child sex (125 boys and 96 girls) when did not adjust for folic acid supplementation in pregnant women.

| APs | VCI | VSI | WMI | FRI | PSI | FSIQ |
| --- | --- | --- | --- | --- | --- | --- |
|  | *β (95%CI)* | *β (95%CI)* | *β (95%CI)* | *β (95%CI)* | *β (95%CI)* | *β (95%CI)* |
| Total |  |  |  |  |  |  |
| NP (cont) | -1.77 (-3.92,0.38) | -1.82 (-4.24,0.60) | -1.97 (-4.66,0.72) | -0.16 (-3.32,3.00) | -1.19 (-4.49,2.10) | -2.39 (-4.58,-0.20)* |
| Tertile 1 | Ref | Ref | Ref | Ref | Ref | Ref |
| Tertile 2 | -1.20 (-4.77,2.36) | 1.50 (-2.49,5.50) | 2.12 (-2.33,6.57) | 3.95 (-0.24,8.14)# | -0.64 (-5.10,3.82) | 0.08 (-3.56,3.73) |
| Tertile 3 | -0.71 (-4.34,2.93) | -0.63 (-4.70,3.45) | -0.33 (-4.86,4.2) | -1.01 (-5.49,3.46) | -1.11 (-5.88,3.65) | -1.14 (-4.85,2.58) |
| *P* for trend | 0.729 | 0.709 | 0.824 | 0.723 | 0.642 | 0.527 |
| 4-N-NP(cont) | 0.90 (-5.79,7.60) | -2.98 (-10.49,4.53) | 2.48 (-5.89,10.84) | 3.70 (-5.16,12.56) | 3.39 (-5.88,12.66) | 0.73 (-6.12,7.57) |
| Tertile 1 | Ref | Ref | Ref | Ref | Ref | Ref |
| Tertile 2 | -2.10 (-5.59,1.39) | -1.76 (-5.70,2.18) | -1.66 (-6.04,2.73) | -0.45 (-4.69,3.78) | 0.50 (-4.01,5.01) | -2.86 (-6.41,0.70) |
| Tertile 3 | 0.78 (-2.40,3.95) | -0.25 (-3.83,3.33) | 0.25 (-3.73,4.23) | 4.69 (0.89,8.50)* | 2.47 (-1.58,6.52) | 1.01 (-2.22,4.24) |
| *P* for trend | 0.682 | 0.858 | 0.934 | 0.021 | 0.238 | 0.604 |
| 4-T-OP (cont) | -0.05 (-1.51,1.40) | 1.16 (-0.47,2.79) | -0.37 (-2.19,1.45) | -0.23 (-1.98,1.53) | -0.29 (-2.12,1.55) | -0.12 (-1.61,1.37) |
| Tertile 1 | Ref | Ref | Ref | Ref | Ref | Ref |
| Tertile 2 | -0.80 (-4.31,2.70) | -2.38 (-6.27,1.52) | -5.18 (-9.51,-0.85)* | -1.32 (-5.66,3.02) | 3.26 (-1.25,7.77) | -1.24 (-4.82,2.34) |
| Tertile 3 | 1.47 (-2.06,5.00) | 2.21 (-1.72,6.14) | -1.01 (-5.37,3.36) | -0.28 (-4.67,4.11) | 1.80 (-2.76,6.37) | 1.07 (-2.54,4.68) |
| *P* for trend | 0.621 | 0.234 | 0.734 | 0.909 | 0.452 | 0.526 |
| 4-N-OP(cont) | 1.90 (-3.83,7.63) | 4.26 (-2.16,10.67) | -0.54 (-7.71,6.63) | 3.56 (-2.85,9.96) | 0.78 (-5.94,7.49) | 3.05 (-2.80,8.90) |
| Tertile 1 | Ref | Ref | Ref | Ref | Ref | Ref |
| Tertile 2 | 0.02 (-3.38,3.43) | 0.75 (-3.07,4.58) | -0.72 (-4.98,3.53) | 2.12 (-2.15,6.39) | 2.41 (-2.06,6.87) | -0.80 (-4.28,2.68) |
| Tertile 3 | 0.83 (-2.45,4.11) | 0.34 (-3.35,4.02) | -1.15 (-5.25,2.95) | 2.56 (-1.53,6.65) | -0.57 (-4.85,3.71) | 0.21 (-3.14,3.57) |
| *P* for trend | 0.387 | 0.852 | 0.579 | 0.207 | 0.857 | 0.912 |
| Boys |  |  |  |  |  |  |
| NP (cont) | -1.45 (-4.60,1.70) | -0.86 (-4.16,2.45) | -1.02 (-4.88,2.84) | -1.57 (-5.77,2.63)# | -1.19 (-5.26,2.87) | -1.65 (-4.88,1.58) |
| Tertile 1 | Ref | Ref | Ref | Ref | Ref | Ref |
| Tertile 2 | -0.12 (-5.36,5.11) | 2.64 (-2.81,8.09) | 2.02 (-4.37,8.41) | 5.30 (-0.65,11.25) | 0.83 (-5.13,6.79) | 0.27 (-5.10,5.64) |
| Tertile 3 | 0.18 (-5.24,5.59) | -0.22 (-5.85,5.41) | 0.17 (-6.43,6.77) | -3.63 (-10.08,2.81) | -3.44 (-9.90,3.02) | -1.02 (-6.57,4.54) |
| *P* for trend | 0.940 | 0.834 | 0.977 | 0.405 | 0.333 | 0.687 |
| 4-N-NP(cont) | 3.81 (-5.15,12.77) | -5.14 (-14.51,4.22) | 0.96 (-10.03,11.95) | 2.99 (-10.57,16.54) | 11.34 (-1.54,24.21)# | 1.43 (-7.80,10.65) |
| Tertile 1 | Ref | Ref | Ref | Ref | Ref | Ref |
| Tertile 2 | -2.74 (-8.01,2.53) | -3.35 (-8.91,2.21) | -3.68 (-10.17,2.82) | -4.97 (-11.42,1.49) | -1.15 (-7.49,5.19) | -4.4 (-9.76,0.96) |
| Tertile 3 | 2.10 (-2.23,6.43) | -1.81 (-6.38,2.76) | 0.15 (-5.19,5.48) | 3.24 (-2.03,8.50) | 3.93 (-1.24,9.10) | 1.91 (-2.49,6.32) |
| *P* for trend | 0.940 | 0.437 | 0.951 | 0.293 | 0.152 | 0.393 |
| 4-T-OP (cont) | 0.52 (-1.55,2.59) | 1.62 (-0.53,3.77) | -0.18 (-2.72,2.35) | -0.66 (-3.26,1.95) | -0.66 (-3.18,1.85) | 0.20 (-1.93,2.33) |
| Tertile 1 | Ref | Ref | Ref | Ref | Ref | Ref |
| Tertile 2 | -0.16 (-5.19,4.87) | -1.45 (-6.72,3.82) | -4.88 (-11.01,1.25) | -2.89 (-9.38,3.6) | 3.36 (-2.87,9.59) | -0.93 (-6.12,4.27) |
| Tertile 3 | 2.94 (-1.97,7.86) | 2.11 (-3.04,7.25) | -1.19 (-7.18,4.80) | -2.41 (-8.84,4.03) | -0.54 (-6.72,5.64) | 1.09 (-3.99,6.16) |
| *P* for trend | 0.224 | 0.393 | 0.746 | 0.474 | 0.811 | 0.652 |
| 4-N-OP(cont) | 2.78 (-6.03,11.59) | 2.34 (-6.89,11.57) | 0.38 (-10.41,11.17) | 0.42 (-9.64,10.48) | 7.38 (-2.20,16.97) | 5.21 (-3.80,14.22) |
| Tertile 1 | Ref | Ref | Ref | Ref | Ref | Ref |
| Tertile 2 | 1.92 (-3.06,6.89) | 1.31 (-3.89,6.51) | -0.51 (-6.58,5.56) | -1.55 (-7.93,4.82) | 2.60 (-3.53,8.74) | 0.30 (-4.83,5.42) |
| Tertile 3 | 0.59 (-4.00,5.18) | -1.24 (-6.04,3.56) | -2.84 (-8.44,2.76) | 0.58 (-5.28,6.44) | -0.45 (-6.10,5.19) | -0.19 (-4.92,4.54) |
| *P* for trend | 0.807 | 0.600 | 0.313 | 0.872 | 0.917 | 0.934 |
| Girls |  |  |  |  |  |  |
| NP (cont) | -1.99 (-5.20,1.21) | -1.79 (-5.60,2.02) | 3.29 (-1.92,8.50) | -3.38 (-6.96,0.21)# | -1.63 (-7.63,4.38) | -2.89 (-6.07,0.28)# |
| Tertile 1 | Ref | Ref | Ref | Ref | Ref | Ref |
| Tertile 2 | -2.48 (-7.79,2.84) | 2.36 (-3.96,8.67) | 1.55 (-4.49,7.58) | 4.78 (-1.50,11.05) | -2.78 (-10.05,4.48) | 0.45 (-4.90,5.79) |
| Tertile 3 | -1.25 (-6.63,4.14) | 1.24 (-5.16,7.64) | -0.57 (-6.66,5.53) | 1.92 (-4.83,8.67) | -0.50 (-8.31,7.31) | -0.42 (-5.83,4.99) |
| *P* for trend | 0.662 | 0.713 | 0.834 | 0.580 | 0.901 | 0.870 |
| 4-N-NP(cont) | -3.37 (-14.77,8.03) | 1.65 (-11.89,15.19) | 6.11 (-6.44,18.66) | 13.04 (0.47,25.61)* | -0.67 (-15.11,13.76) | 1.45 (-9.98,12.87) |
| Tertile 1 | Ref | Ref | Ref | Ref | Ref | Ref |
| Tertile 2 | -2.02 (-6.97,2.93) | -1.13 (-7.00,40.73) | 0.50 (-5.07,6.07) | 4.11 (-1.85,10.07) | 2.66 (-4.38,9.70) | -1.80 (-6.76,3.15) |
| Tertile 3 | -1.00 (-6.09,4.08) | 1.79 (-4.23,7.82) | 2.61 (-3.16,8.38) | 6.46 (0.73,12.19)* | 1.71 (-5.06,8.47) | -0.12 (-5.21,4.97) |
| *P* for trend | 0.637 | 0.611 | 0.385 | 0.025 | 0.590 | 0.893 |
| 4-T-OP (cont) | -0.70 (-2.88,1.49) | 1.12 (-1.47,3.71) | 0.46 (-2.09,3.02) | -0.56 (-3.03,1.91) | 0.26 (-2.66,3.17) | -0.47 (-2.66,1.72) |
| Tertile 1 | Ref | Ref | Ref | Ref | Ref | Ref |
| Tertile 2 | -1.46 (-6.61,3.69) | -3.26 (-9.19,2.67) | -4.58 (-10.39,1.23) | -0.33 (-6.46,5.79) | 2.44 (-4.55,9.43) | -1.51 (-6.64,3.62) |
| Tertile 3 | -0.70 (-6.13,4.73) | 3.57 (-2.69,9.82) | -0.19 (-6.21,5.82) | 3.11 (-3.22,9.45) | 4.48 (-2.75,11.71) | 1.16 (-4.24,6.57) |
| *P* for trend | 0.807 | 0.249 | 0.995 | 0.340 | 0.216 | 0.653 |
| 4-N-OP(cont) | 2.91 (-5.29,11.12) | 8.99 (-0.58,18.55) | 8.57 (-0.46,17.61)# | -0.18 (-9.46,9.10) | -4.13 (-14.67,6.42) | 3.07 (-5.14,11.27) |
| Tertile 1 | Ref | Ref | Ref | Ref | Ref | Ref |
| Tertile 2 | -1.32 (-6.35,3.71) | 0.76 (-5.20,6.73) | -0.41 (-6.13,5.32) | 6.16 (0.06,12.25)* | 1.99 (-5.22,9.20) | -1.32 (-6.36,3.72) |
| Tertile 3 | 2.03 (-3.06,7.12) | 3.61 (-2.43,9.65) | 1.53 (-4.26,7.33) | 5.55 (-0.61,11.71)# | -0.60 (-7.88,6.68) | 1.73 (-3.37,6.83) |
| *P* for trend | 0.478 | 0.246 | 0.622 | 0.055 | 0.935 | 0.551 |

Abbreviations: 95%CI, 95% confidence interval; VCI, verbal comprehension index; VSI, the visual space index; FRI, the fluid reasoning index; WMI, the working memory index; PSI, processing speed index; FSIQ, full-scale intelligence quotient; APs, Alkylphenols; NP, Nonylphenol; 4-N-NP, 4-Nonylphenol; 4-T-OP, 4-tert-Octylphenol; 4-N-OP, 4-n-Octylphenol.

The models were adjusted for maternal age at delivery, maternal pre-pregnancy BMI, passive smoking, maternal education, household income, folic acid supplementation,

breastfeeding duration, child age, and child sex (except for sex stratification).

^⁎^ *P* < 0.05；^#^ *P* < 0.10.


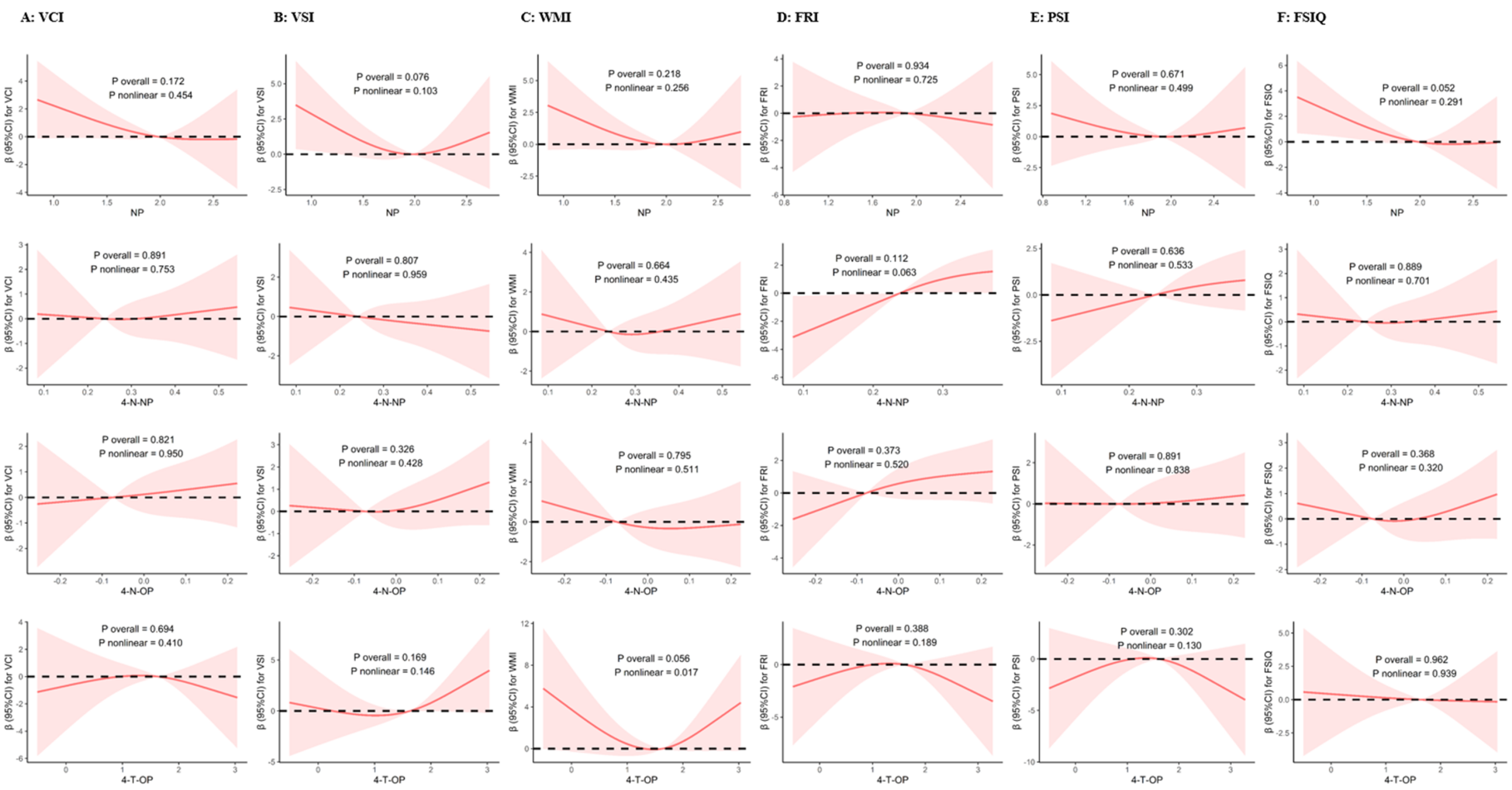


**Fig. S1** Restricted cubic spline (RCS) models for log10-transformed APs associated with childhood intelligence quotient with knots at the 10th, 50th, and 90th percentiles in total children. The red lines with shading represent adjust β (95% CI) based on RCS, and black dotted lines represent the null. All models were adjusted for maternal age at delivery, maternal pre-pregnancy BMI, passive smoking, maternal education, household income, folic acid supplementation, breastfeeding duration, child age, and child sex.


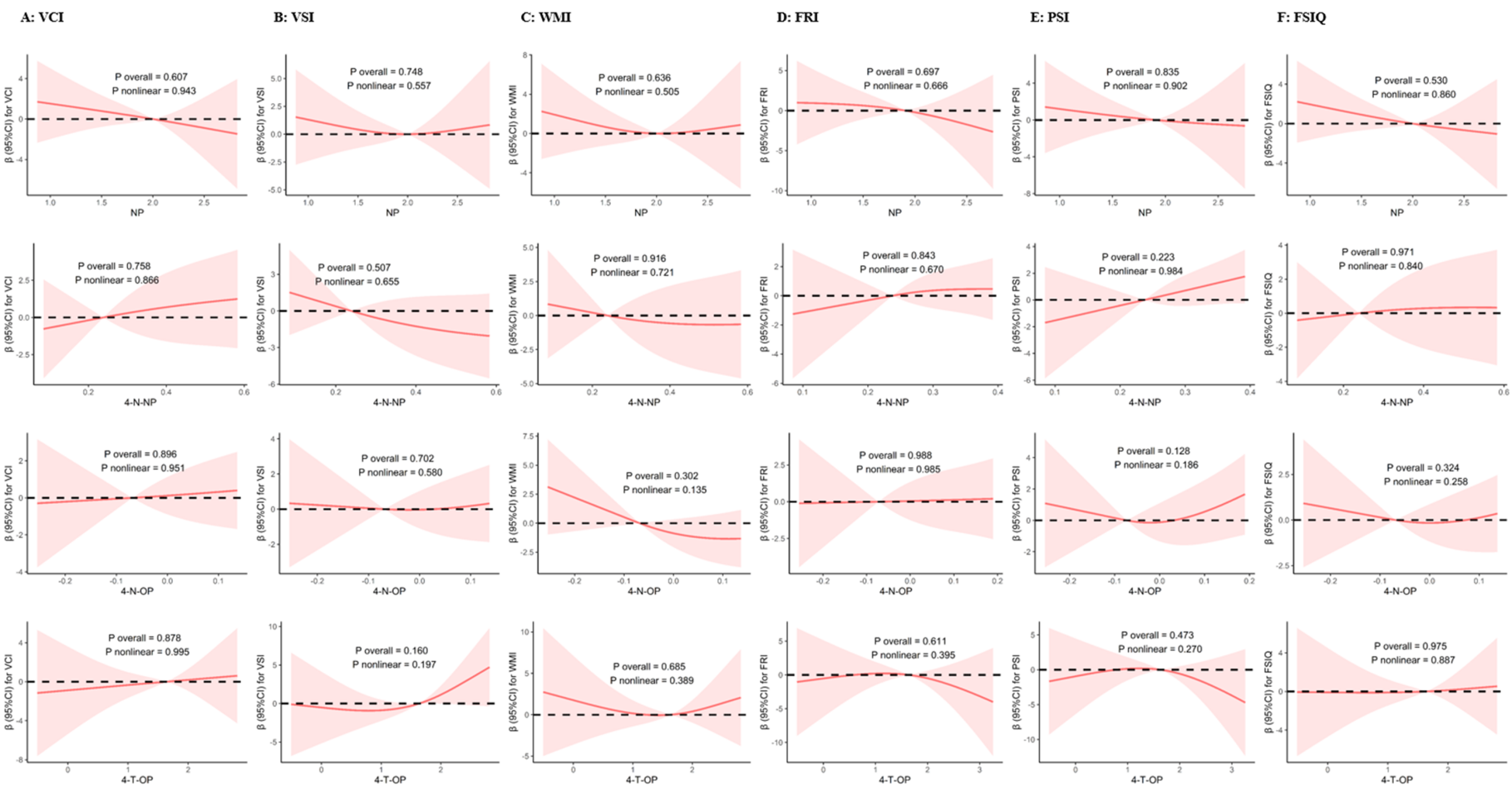


**Fig. S2** Restricted cubic spline (RCS) models for log10-transformed APs associated with childhood intelligence quotient with knots at the 10th, 50th, and 90th percentiles in boys. The red lines with shading represent adjust β (95% CI) based on RCS, and black dotted lines represent the null. All models were adjusted for maternal age at delivery, maternal pre-pregnancy BMI, passive smoking, maternal education, household income, folic acid supplementation, breastfeeding duration, and child age.


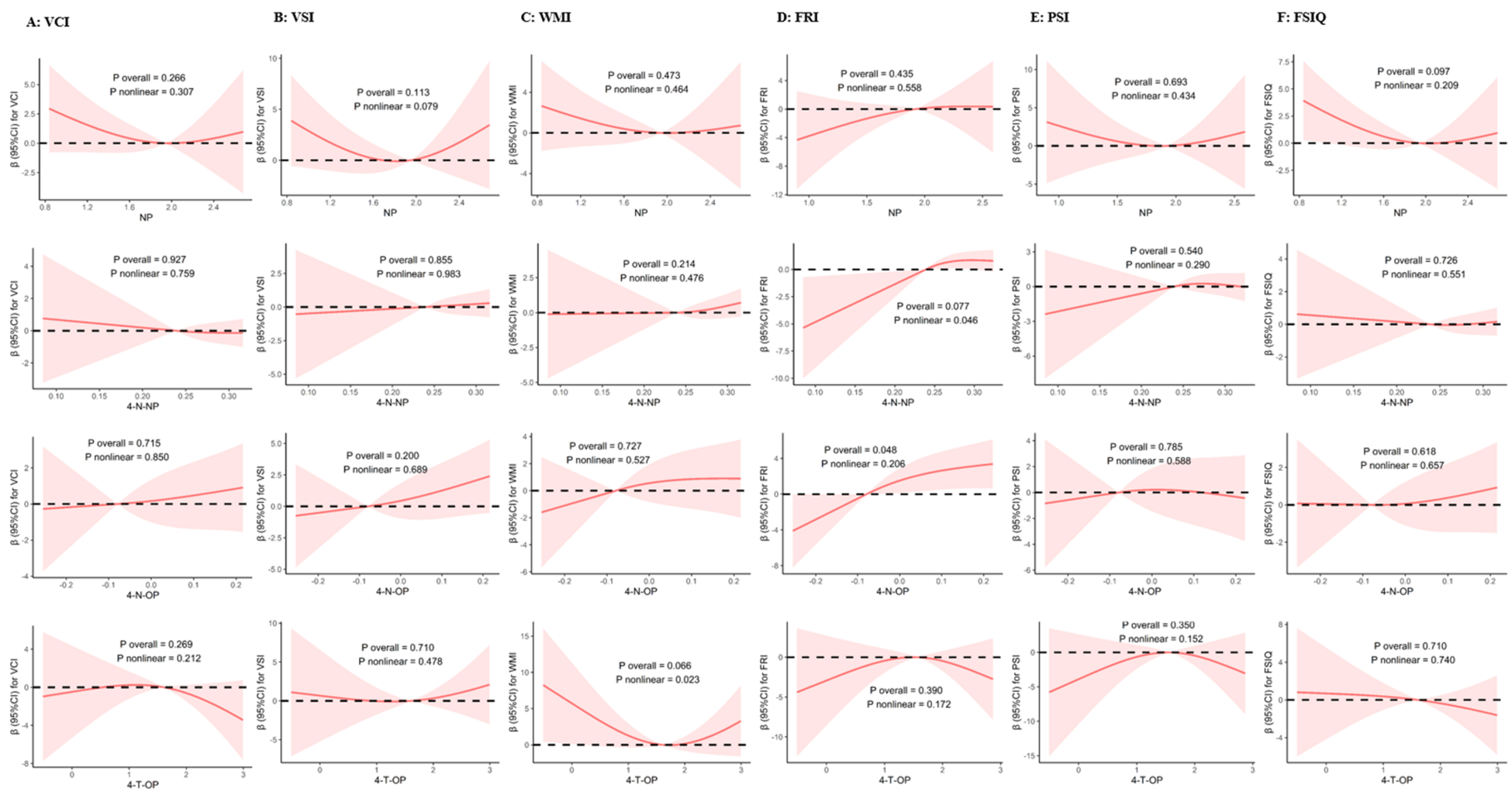


**Fig. S3** Restricted cubic spline (RCS) models for log10-transformed APs associated with childhood intelligence quotient with knots at the 10th, 50th, and 90th percentiles in girls. The red lines with shading represent adjust β (95% CI) based on RCS, and black dotted lines represent the null. All models were adjusted for maternal age at delivery, maternal pre-pregnancy BMI, passive smoking, maternal education, household income, folic acid supplementation, breastfeeding duration, and child age.


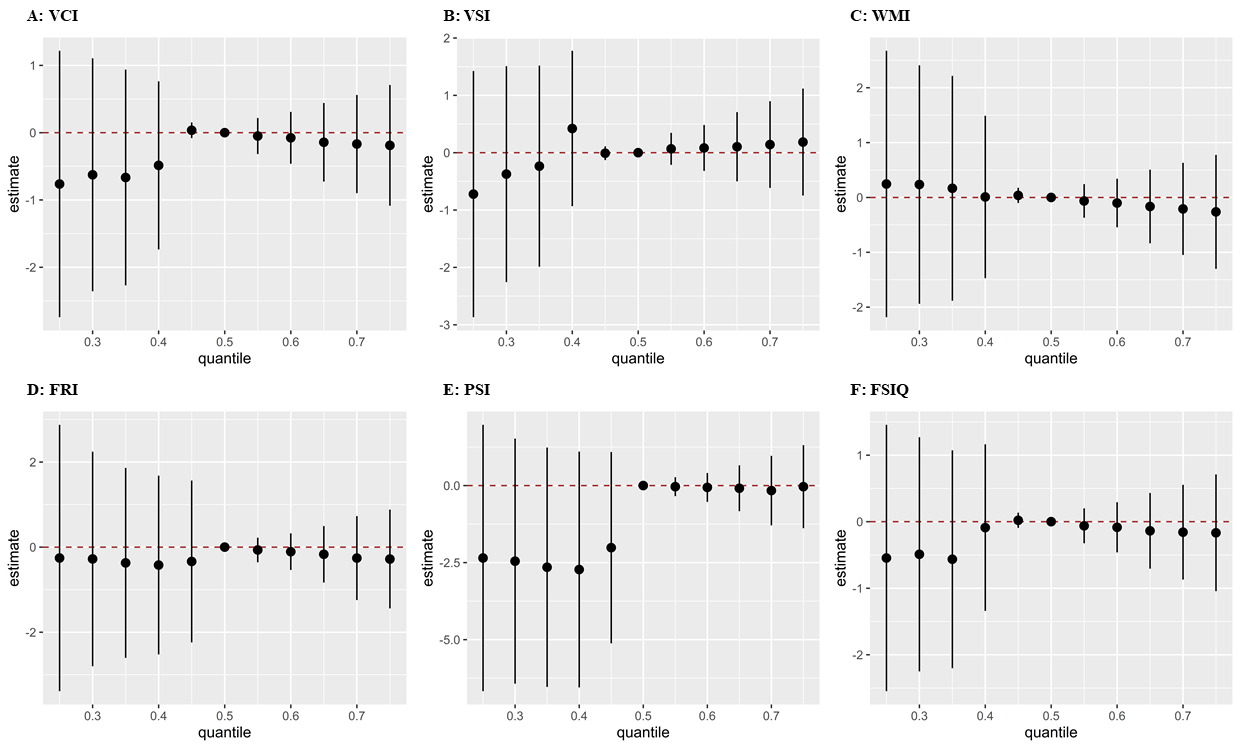


**Fig. S4** Combined effect of the maternal serum APs on childhood intelligence quotient in boys. APs were log10- transformed. Black circle indicates effect estimates, black vertical lines represent 95% confidence intervals, and red dotted lines represent the null. All models were adjusted for maternal age at delivery, maternal pre-pregnancy BMI, passive smoking, maternal education, household income, folic acid supplementation, breastfeeding duration, and child age.


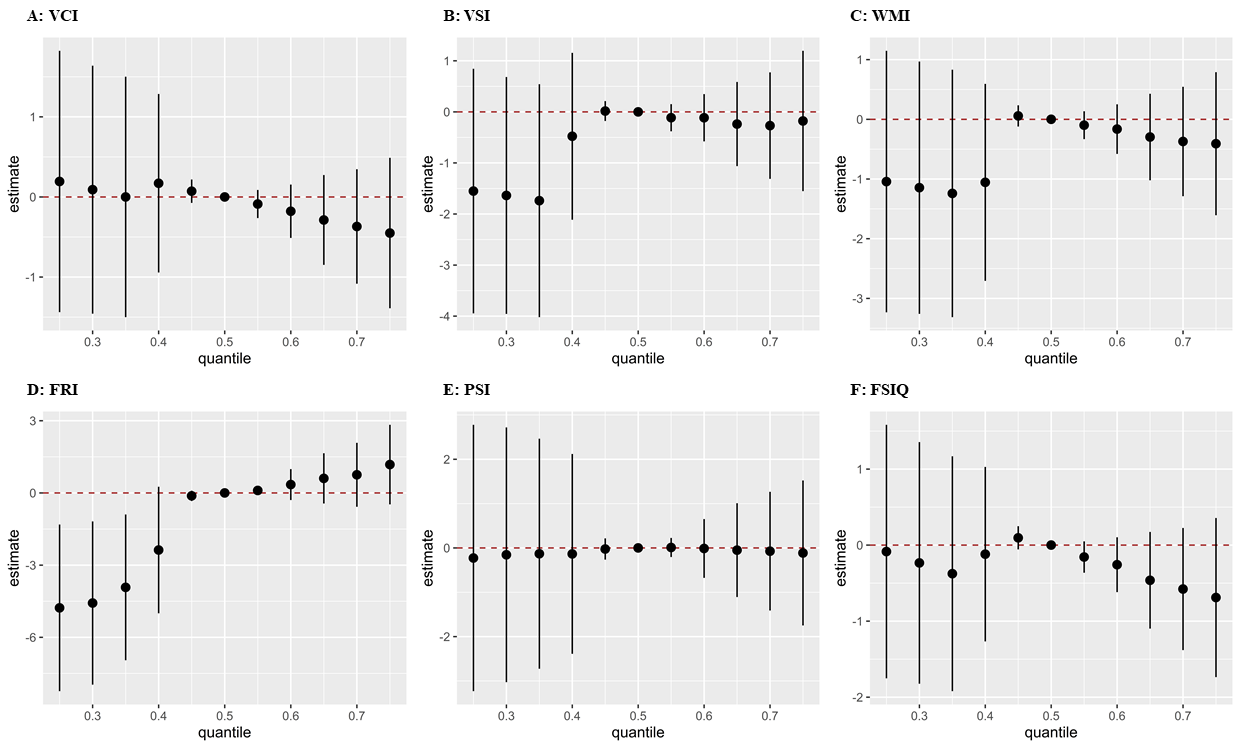


**Fig. S5** Combined effect of the maternal serum APs on childhood intelligence quotient in girls. APs were log10- transformed. Black circle indicates effect estimates, black vertical lines represent 95% confidence intervals, and red dotted lines represent the null. All models were adjusted for maternal age at delivery, maternal pre-pregnancy BMI, passive smoking, maternal education, household income, folic acid supplementation, breastfeeding duration, and child age.

**
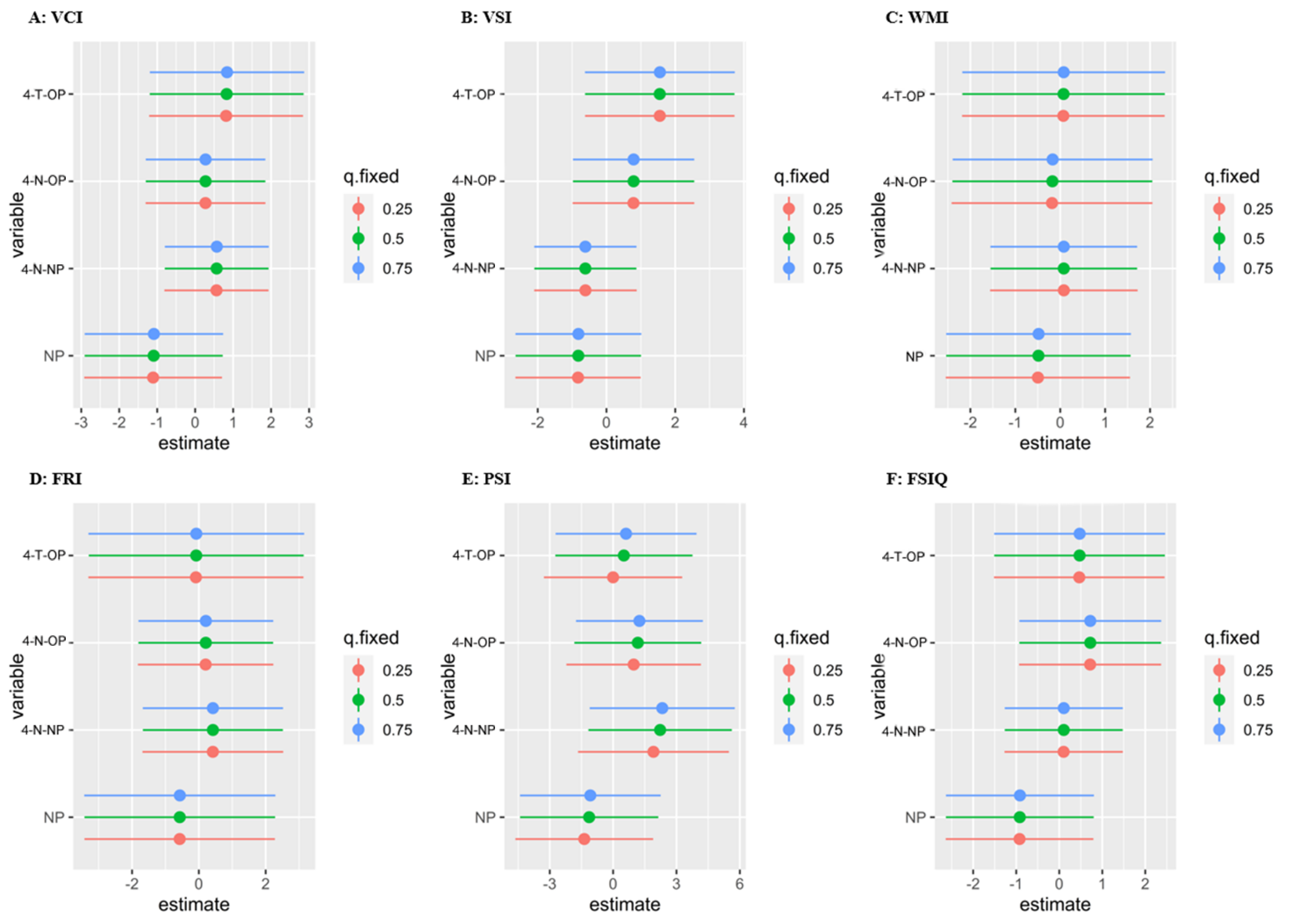
**

**Fig. S6** Association (estimates and 95% confidence intervals) of each maternal serum AP with childhood intelligence quotient in boys, when the other maternal APs were fixed at their 25th, 50th, and 75th percentiles. All models were adjusted for maternal age at delivery, maternal pre-pregnancy BMI, passive smoking, maternal education, household income, folic acid supplementation, breastfeeding duration, and child age.

**
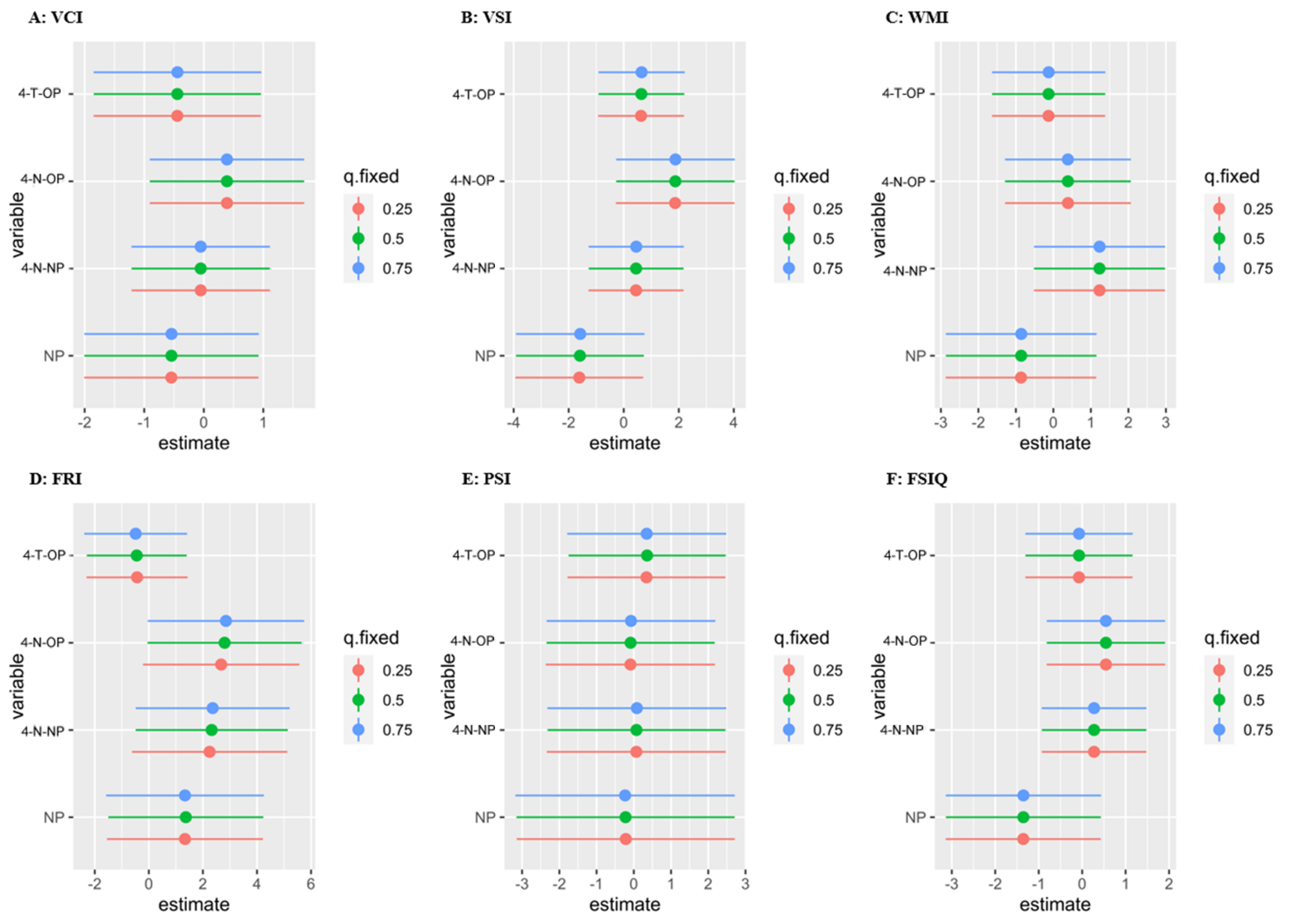
**

**Fig. S7** Association (estimates and 95% confidence intervals) of each maternal serum AP with childhood intelligence quotient in gilrs, when the other APs were fixed at their 25th, 50th, and 75th percentiles. All models were adjusted for maternal age at delivery, maternal pre-pregnancy BMI, passive smoking, maternal education, household income, folic acid supplementation, breastfeeding duration, and child age.

**
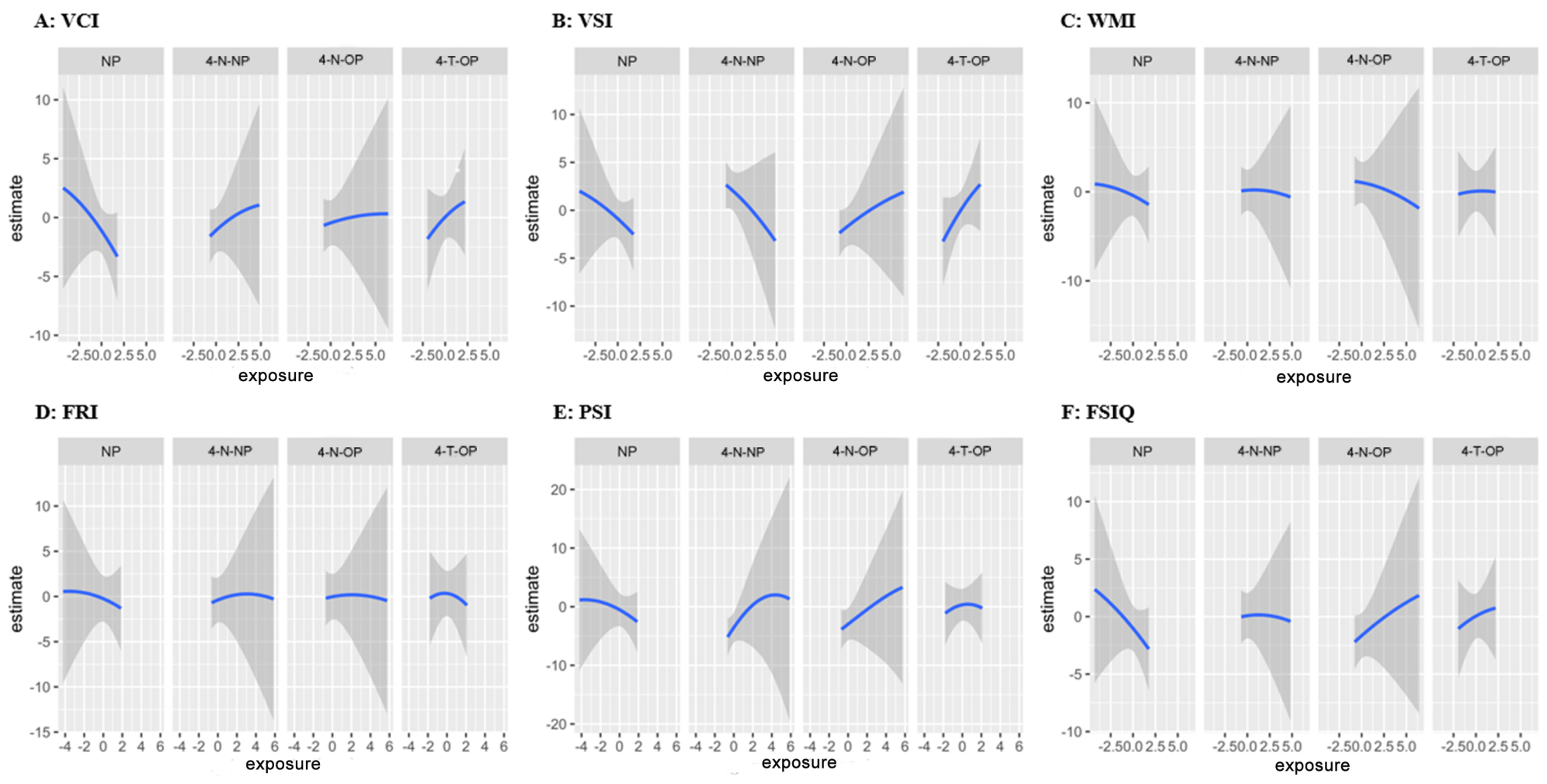
**

**Fig. S8** Univariate dose-response function (95% confidence intervals) between the log10-transformed concentrations of per maternal serum APs and childhood intelligence quotient, when fixing the concentrations of other APs at the 50th percentile in boys. Models were adjusted for were adjusted for maternal age at delivery, maternal pre-pregnancy BMI, passive smoking, maternal education, household income, folic acid supplementation, breastfeeding duration, and child age.

**
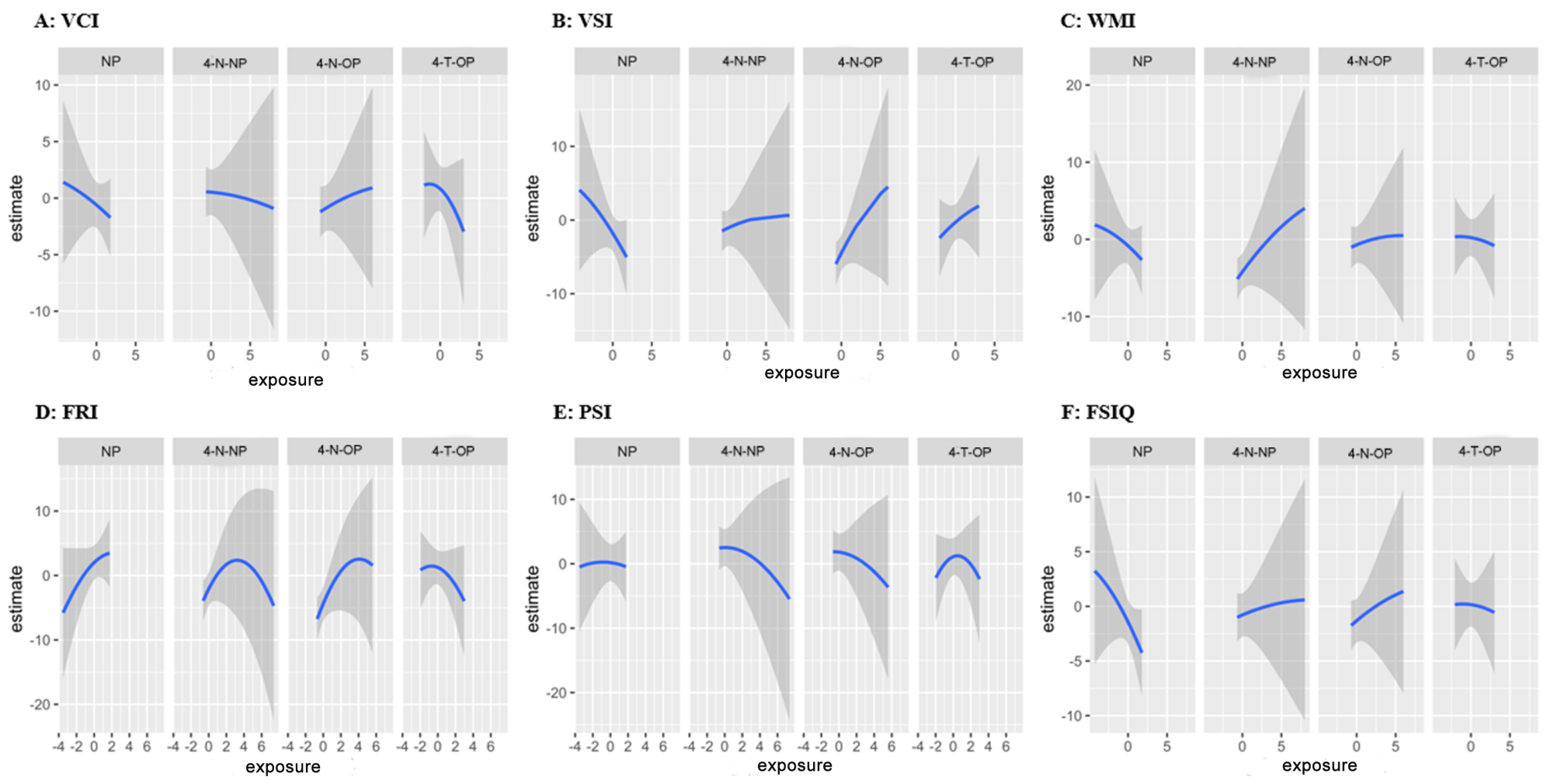
**

**Fig. S9** Univariate dose-response function (95% confidence intervals) between the log10-transformed concentrations of per maternal serum APs and childhood intelligence quotient, when fixing the concentrations of other APs at the 50th percentile in girls. Models were adjusted for were adjusted for maternal age at delivery, maternal pre-pregnancy BMI, passive smoking, maternal education, household income, folic acid supplementation, breastfeeding duration, and child age.


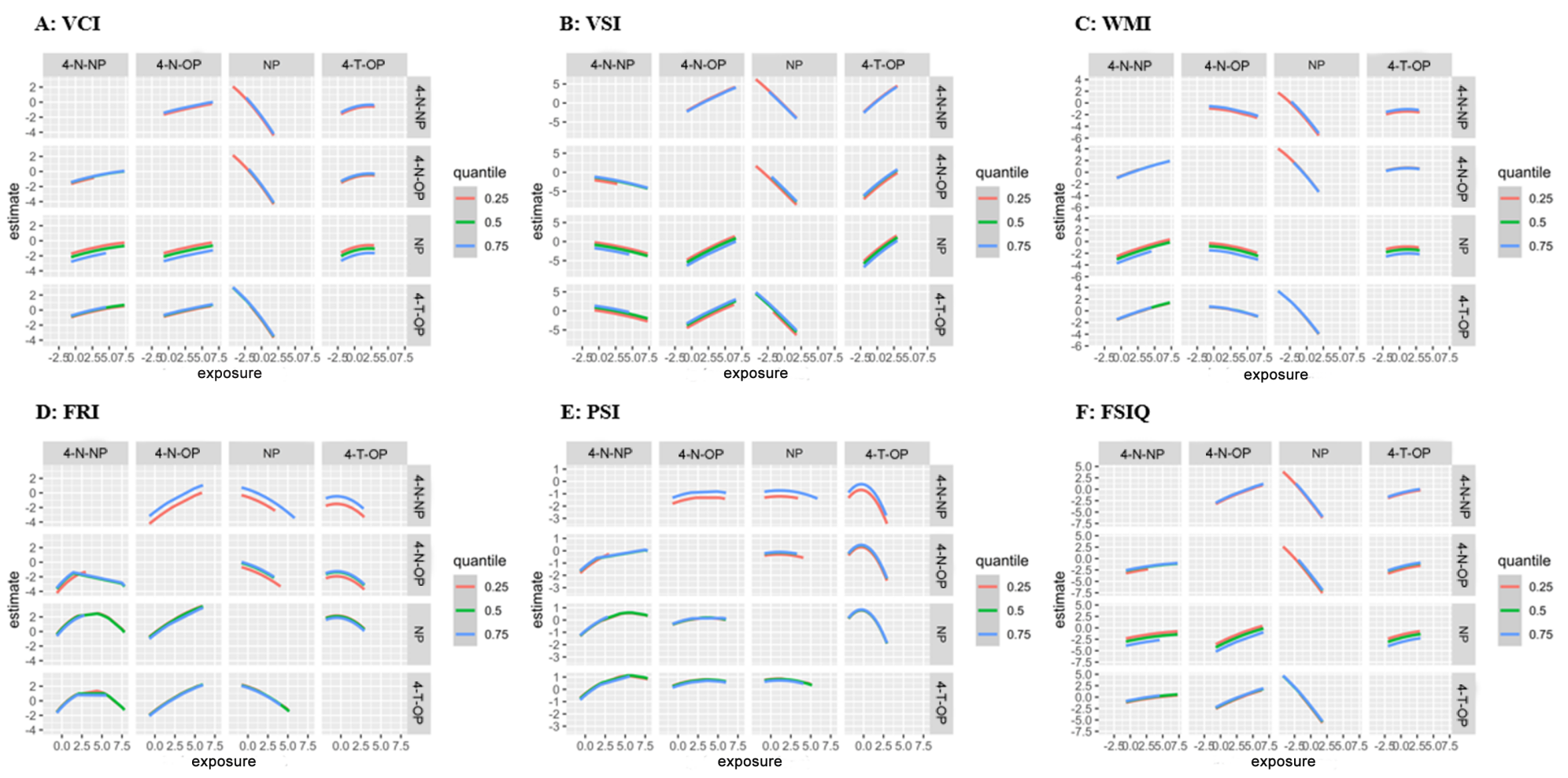


**Fig. S10** Bivariate exposure-response function between each pair of maternal serum APs and childhood intelligence quotient, when fixing the other APs at their 25th, 50th, and 75th percentiles in total children. Models were adjusted for were adjusted for maternal age at delivery, maternal pre-pregnancy BMI, passive smoking, maternal education, household income, folic acid supplementation, breastfeeding duration, child age, and child sex.


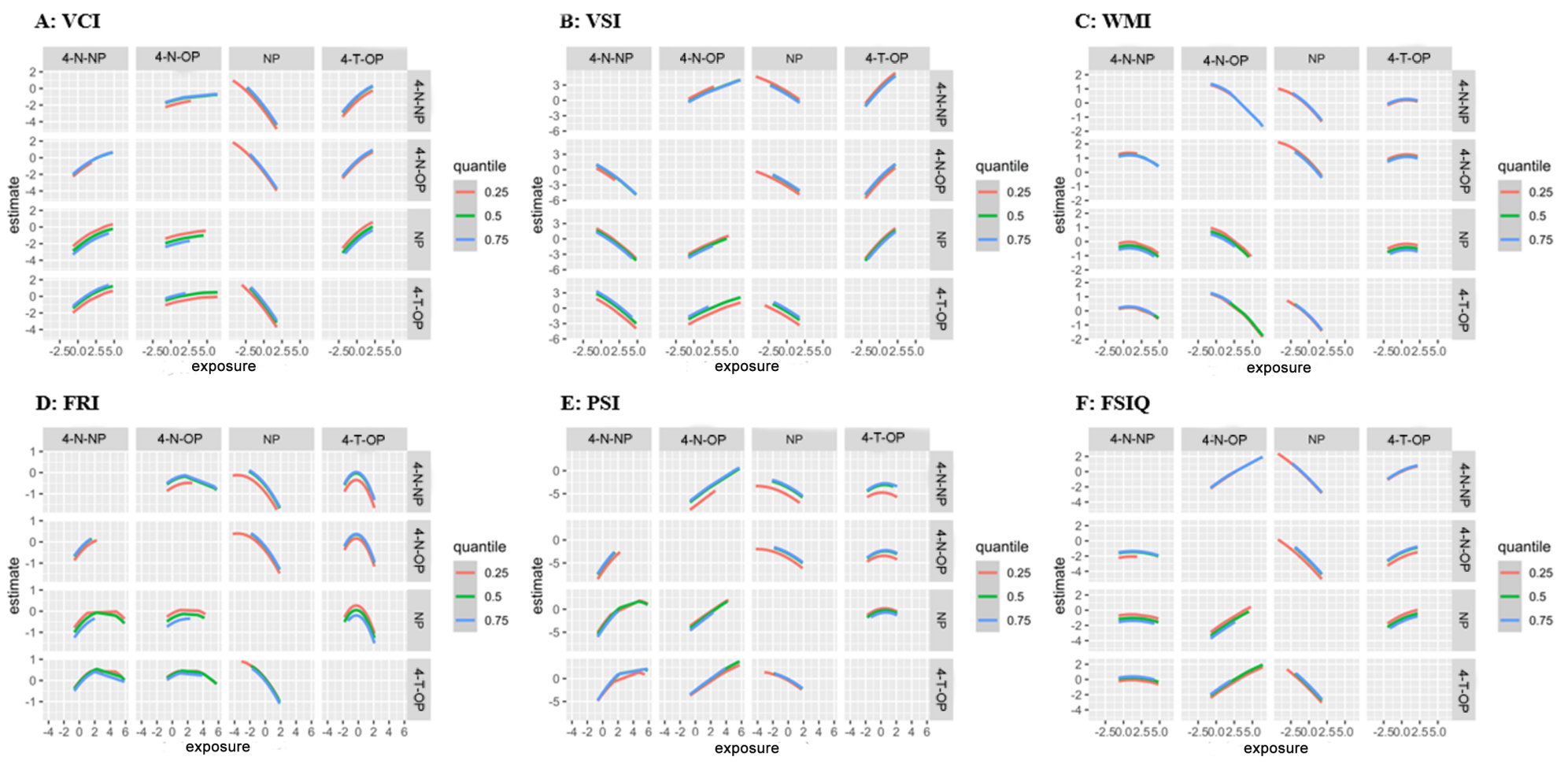


**Fig. S11** Bivariate exposure-response function between each pair of maternal serum APs and childhood intelligence quotient, when fixing the other APs at their 25th, 50th, and 75th percentiles in boys. Models were adjusted for were adjusted for maternal age at delivery, maternal pre-pregnancy BMI, passive smoking, maternal education, household income, folic acid supplementation, breastfeeding duration, and child age.


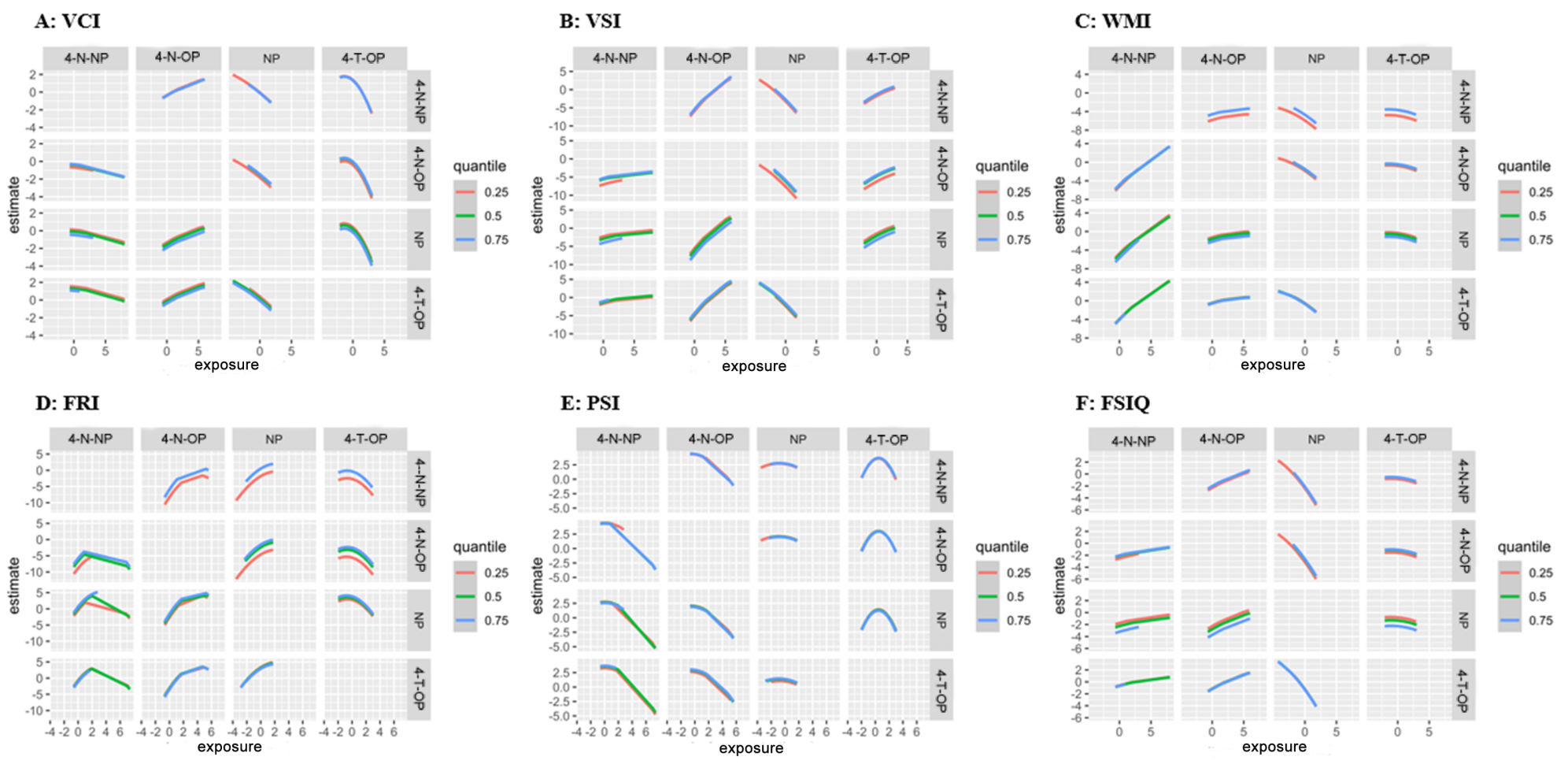


**Fig. S12** Bivariate exposure-response function between each pair of maternal serum APs and childhood intelligence quotient, when fixing the other APs at their 25th, 50th, and 75th percentiles in girls. Models were adjusted for were adjusted for maternal age at delivery, maternal pre-pregnancy BMI, passive smoking, maternal education, household income, folic acid supplementation, breastfeeding duration, and child age.
